# Is IQG-607 a Potential Metallodrug or Metallopro-Drug With a Defined Molecular Target in *Mycobacterium tuberculosis*?

**DOI:** 10.3389/fmicb.2018.00880

**Published:** 2018-05-01

**Authors:** Bruno L. Abbadi, Valnês da Silva Rodrigues-Junior, Adilio da Silva Dadda, Kenia Pissinate, Anne D. Villela, Maria M. Campos, Luiz G. de França Lopes, Cristiano V. Bizarro, Pablo Machado, Eduardo H. S. Sousa, Luiz A. Basso

**Affiliations:** ^1^Centro de Pesquisas em Biologia Molecular e Funcional, Instituto Nacional de Ciência e Tecnologia em Tuberculose, Pontifícia Universidade Católica do Rio Grande do Sul, Porto Alegre, Brazil; ^2^Programa de Pós-Graduação em Biologia Celular e Molecular, Pontifícia Universidade Católica do Rio Grande do Sul, Porto Alegre, Brazil; ^3^Programa de Pós-Graduação em Medicina e Ciências da Saúde, Pontifícia Universidade Católica do Rio Grande do Sul, Porto Alegre, Brazil; ^4^Grupo de Bioinorgânica, Departamento de Química Orgânica e Inorgânica, Universidade Federal do Ceará, Fortaleza, Brazil

**Keywords:** *Mycobacterium tuberculosis*, pentacyano(isoniazid)ferrate(II) complex, IQG-607, metallodrug, molecular target, isoniazid analog, mode of resistance, bioinorganic chemistry

## Abstract

The emergence of strains of *Mycobacterium tuberculosis* resistant to isoniazid (INH) has underscored the need for the development of new anti-tuberculosis agents. INH is activated by the mycobacterial *katG*-encoded catalase-peroxidase, forming an acylpyridine fragment that is covalently attached to the C4 of NADH. This isonicotinyl-NAD adduct inhibits the activity of 2-*trans*-enoyl-ACP(CoA) reductase (InhA), which plays a role in mycolic acid biosynthesis. A metal-based INH analog, Na_3_[Fe^II^(CN)_5_(INH)]·4H_2_O, IQG-607, was designed to have an electronic redistribution on INH moiety that would lead to an intramolecular electron transfer to bypass KatG activation. HPLC and EPR studies showed that the INH moiety can be oxidized by superoxide or peroxide yielding similar metabolites and isonicotinoyl radical only when associated to IQG-607, thereby supporting redox-mediated drug activation as a possible mechanism of action. However, IQG-607 was shown to inhibit the *in vitro* activity of both wild-type and INH-resistant mutant InhA enzymes in the absence of KatG activation. IQG-607 given by the oral route to *M. tuberculosis*-infected mice reduced lung lesions. Experiments using early and late controls of infection revealed a bactericidal activity for IQG-607. HPLC and voltammetric methods were developed to quantify IQG-607. Pharmacokinetic studies showed short half-life, high clearance, moderate volume of distribution, and low oral bioavailability, which was not altered by feeding. Safety and toxic effects of IQG-607 after acute and 90-day repeated oral administrations in both rats and minipigs showed occurrence of mild to moderate toxic events. Eight multidrug-resistant strains (MDR-TB) were resistant to IQG-607, suggesting an association between *katG* mutation and increasing MIC values. Whole genome sequencing of three spontaneous IQG-607-resistant strains harbored *katG* gene mutations. MIC measurements and macrophage infection experiments with a laboratorial strain showed that *katG* mutation is sufficient to confer resistance to IQG-607 and that the macrophage intracellular environment cannot trigger the self-activation mechanism. Reduced activity of IQG-607 against an *M. tuberculosis* strain overexpressing S94A InhA mutant protein suggested both the need for KatG activation and InhA as its target. Further efforts are suggested to be pursued toward attempting to translate IQG-607 into a chemotherapeutic agent to treat tuberculosis.

## The mode of action of isoniazid (INH) and ethionamide (ETH)

It is estimated that about 4.1% of new tuberculosis (TB) cases are resistant to the leading drugs used in the current treatment (WHO, 2017). In 2016, 600,000 were reported to be new cases resistant to rifampicin only (RR-TB) of which 490,000 were multidrug-resistant TB (MDR-TB) (WHO, 2017). MDR-TB is defined as strains of *Mycobacterium tuberculosis* resistant to at least rifampicin and isoniazid (INH). The occurrence of MDR/RR-TB cases makes the therapy more laborious and reduces its success rate from 85 to 52% (Pai et al., 2016; WHO, 2017). INH mono-resistance is one of the most prevalent type of resistance found in clinical isolates (about 8.5% of all TB cases) (Unissa et al., 2016; WHO, 2017), and it is recognized to significantly increase treatment failure and relapse (Menzies et al., 2009; Gegia et al., 2017). This is of major concern since INH is a widely used first-line anti-TB drug known by the following characteristics: (I) simple molecular structure (a pyridine ring and a hydrazide group); (II) potent early bactericidal activity, capable of killing rapidly-dividing mycobacteria (Jindani et al., 1980); (III) high bioavailability inside the human body; and (IV) narrow range of action, being active only against some species of the genus *Mycobacterium* (Isoniazid, 2008; Unissa et al., 2016).

**Figure 1:**
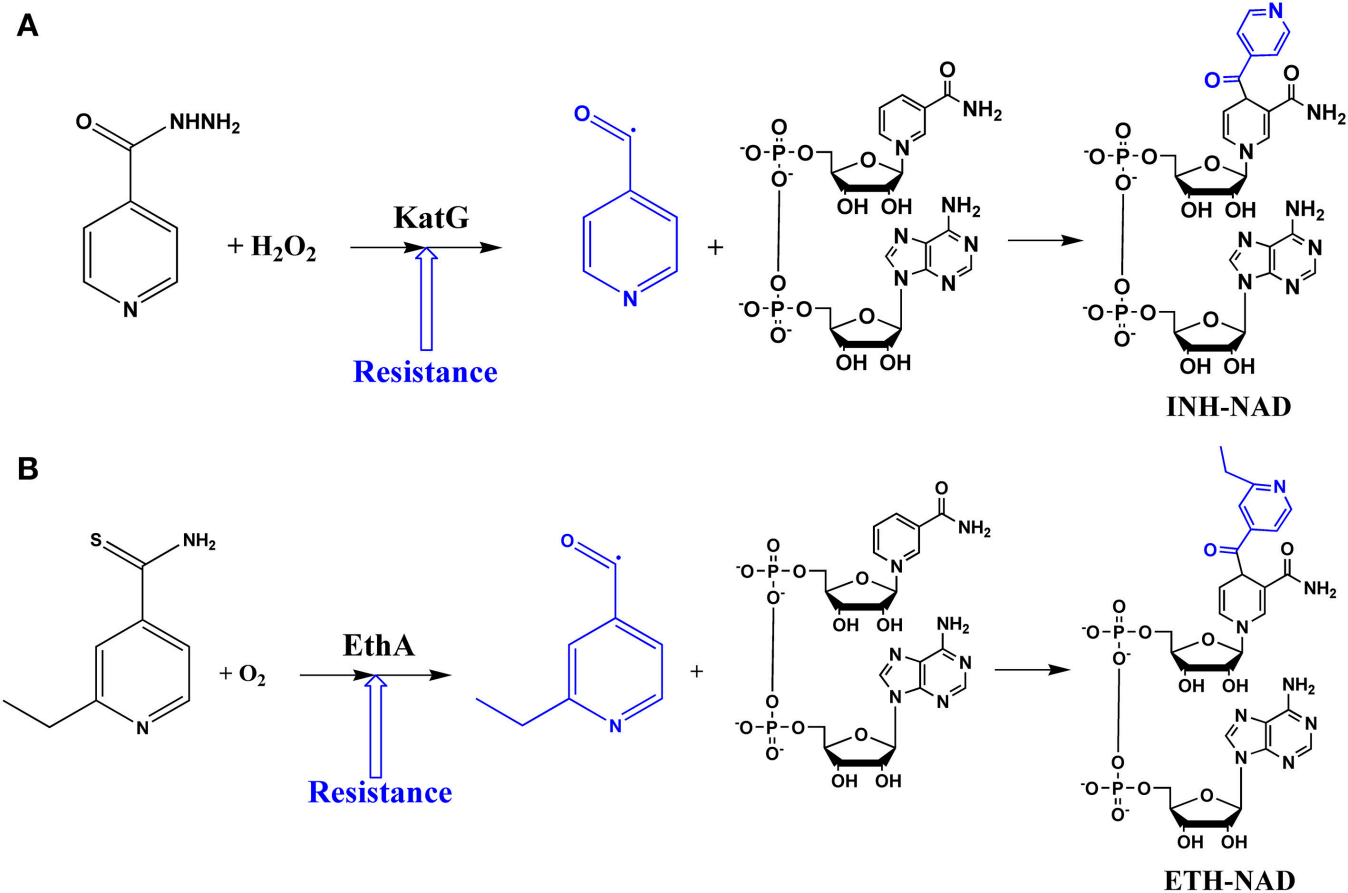
Mechanisms for isoniazid **(A)** and ethionamide **(B)** enzyme-mediated drug activation.

*Mycobacterium tuberculosis* has successfully used two main mechanisms of resistance to avoid death triggered by INH. The alteration of the naturally occurring serine-315 by a threonine in the catalase-peroxidase KatG protein is by far the most widespread mutation found in INH-resistant clinical isolates (up to 94%), followed by the C(-15)T mutation in the *inhA* promoter sequence (Cohen et al., 2014; Vilchèze and Jacobs, 2014). The S315T KatG mutation results in loss of INH activation and ensuing drug resistance (MIC of 5–10 μg/mL) as a result of an insufficient INH-NAD adduct formation (van Soolingen et al., 2000; Suarez et al., 2009), while maintaining the catalase-peroxidase activity necessary for its biological protective role (Wengenack et al., 1997). The *inhA*-promoter C(-15)T mutation is known to cause an increase in the InhA enzyme expression levels, leading to a titration effect by which more compound is needed to inhibit the enzyme and disrupt the mycolic acid biosynthesis (Larsen et al., 2002; Cohen et al., 2014; Vilchèze and Jacobs, 2014). This titration effect leads up to an eight-fold increase in the INH's MIC values, which is considered to be a low level of INH resistance (Vilchèze and Jacobs, 2014). The product of the *M. tuberculosis inhA* gene (InhA) was identified as an NADH-dependent enoyl-ACP (acyl carrier protein) reductase enzyme, which exhibits specificity for long-chain (C_18_ > C_16_) enoyl thioester substrates (Quémard et al., 1995). InhA is a member of the mycobacterial type II fatty acid synthase system (FAS-II), which elongates acyl fatty acid precursors yielding the long carbon chain of the meromycolate branch of mycolic acids, the hallmark of mycobacteria (Schroeder et al., 2002). Mycolic acids are high-molecular-weight α-alkyl, β-hydroxy fatty acids, which appear mostly as bound esters in tetramycolylpentaarabinosyl clusters in the mycobacterial cell wall. The mycobacterial cell wall is comprised of three covalently linked macromolecules: peptidoglycan, arabinogalactan, and mycolic acid (Schroeder et al., 2002). The INH-NAD adduct formed by KatG is a slow, tight-binding competitive inhibitor of InhA enzyme activity with an overall dissociation constant (K_i_^*^) value of 0.75 × 10^−9^ mol L^−1^ (Rawat et al., 2003). INH is a prodrug activated by KatG to form the INH-NAD adduct that inhibits InhA enzyme activity, which results in impaired mycolic acid biosynthesis and ensuing cell death (Figure 1A).

Ethionamide (ETH; 2-ethylthioisonicotinamide), a structural analog of INH, is also a prodrug that requires bioactivation. This process is mediated by the bacterial NADPH-specific flavin adenine dinucleotide–containing monooxygenase (EthA), which is under the control of the transcriptional repressor EthR (Vilchèze and Jacobs, 2014). The active form of ETH reacts with NAD to yield an ETH-NAD adduct, which inhibits InhA, leading to inhibition of mycolic acid biosynthesis (Figure 1B). The mechanisms of resistance to ETH include mutations in genes encoding its activator (*ethA*), its target (*inhA*), or the transcriptional regulator of *ethA* regulator (EthR) (Vilchèze and Jacobs, 2014).

The development of new strategies to combat both pan-sensitive (e.g., to shorten TB treatment course duration) and drug-resistant strains of *M. tuberculosis* is worth pursuing. Our contribution strives to give a brief account of efforts to develop metal-based chemical compounds to overcome redox-mediated mechanism of drug resistance in *M. tuberculosis*. The inorganic chemistry efforts (section Developing New Pharmacological Strategies Using Metals), *in vitro* InhA inhibition studies (section InhA Inhibition by IQG-607), *M. tuberculosis* growth inhibition (section *M. tuberculosis* Growth Inhibition Studies), toxicity (section Toxicity Studies), and pharmacokinetics (section Methods for Quantification and Pharmacokinetic Studies) are presented. In section Elucidation of Mechanism of *M. tuberculosis* Resistance to IQG-607, we believe that the question posed in the title of our manuscript is answered. A few efforts to be pursued further are given in section Perspectives (Nanodelivery Systems or Nano-formulations).

## Developing new pharmacological strategies using metals

A remarkable number of metal-based compounds with potential medical applications have been developed, and some of them approved for clinical use, during the last decades (Barry and Sadler, 2013; Mjos and Orvig, 2014). This heightened interest in inorganic medicinal chemistry is due to both prior successful drugs approved for clinical use (e.g., cisplatin, auranofin, carboplatin, nitroprusside, silver sulfadiazine) (Barry and Sadler, 2013; Mjos and Orvig, 2014) and novel proposals for therapeutic strategies using metal properties (Bruijnincx and Sadler, 2008; Meggers, 2009; Dorr and Meggers, 2014; Yu and Cowan, 2017). Among these unique properties, metal ions can have adjustable redox potential, variable kinetic rates and photochemistry processes, besides wider accessible geometries and stereochemical complexity (e.g., octahedral ≤ 30 isomers) in comparison to carbon-based chemistry (tetrahedral ≤ 2 isomers).

Structural diversity has been explored using metals to create new compounds with a more stringent targeting of biological polymers such as proteins, DNA and RNA (Wilbuer et al., 2010; Meggers, 2011; Sa et al., 2015; Sousa et al., 2017). Some promising examples were reported using staurosporin-like ligand as a scaffold for ruthenium metal complex achieving higher biological activity and improved selectivity toward kinase inhibition (Dorr and Meggers, 2014). The modulation of reactivity promoted by metals has also been explored in the design of donors of either nitric oxide or CO, where ruthenium-based compounds have stood out (Tfouni et al., 2012; Wright and Wright, 2016). Light and electron-transfer triggered processes have been used to generate cytotoxic metal-based compounds with production of reactive oxygen species (ROS), release of NO or CO, and hypoxia-responsive agents (Tfouni et al., 2010; Li et al., 2015; Pires et al., 2016; Romo et al., 2016; Sousa et al., 2016; Abreu et al., 2017; de Sousa et al., 2017). Cobalt(III) complexes, for example, have been used to release drugs or agents for imaging under hypoxia conditions, which enables reduction of Co(III) to Co(II). The latter redox state is labile promoting the release of the active ligand that might be used to target tumors (Graf and Lippard, 2012; Pires et al., 2016). Catalytic-based metallodrugs have also been described, including intracellular reduction of copper(II) complexes that can generate ROS cytotoxic species (Romo et al., 2016; Yu and Cowan, 2017). These compounds have been reported to be active against bacteria and cancer cells (Yu and Cowan, 2017). A few examples of metal-based drugs used in the treatment of different diseases are given in Table 1.

**Table 1:** Some examples of metal-based drugs (Barry and Sadler, 2013; Mjos and Orvig, 2014).

**Metal-based compound**		**Treatment**
Carboplatin **(Pt)**	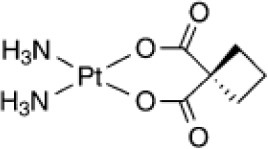	Cancer (approved)
Sodium nitroprusside **(Fe)**	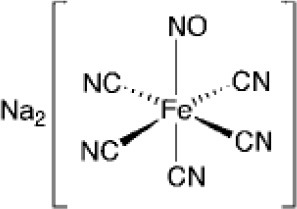	Cardiovascular emergency (approved)
Lithobid **(Li)**	Li_2_(CO_3_)	Bipolar disorder (approved)
Auranofin **(Au)**	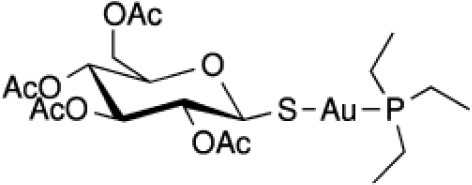	-Arthritis (approved) -Amoebiasis and giardiasis (phase II) -Chronic lymphocytic leukemia (phase II)
Silver sulphadiazine **(Ag)**	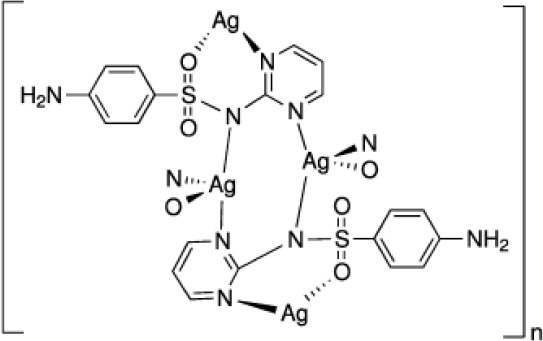	Prevention and treatment of infections in second or third degree burns (approved)
Ferroquine **(Fe)**	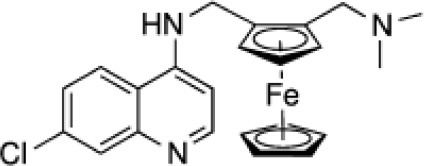	Anti-malarial (phase II)
Fosrenol **(La)**	La_2_(CO_3_)${}_{3}^{.}$4H_2_O	Hyperphosphatemia (approved)
Trisenox **(As)**	As_2_O_3_	Acute promyelocytic leukemia (approved)
Melarsoprol **(As)**	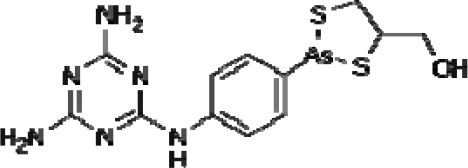	treatment of sleeping sickness (African trypanosomiasis) (approved)
Radiogardase **(Fe)**	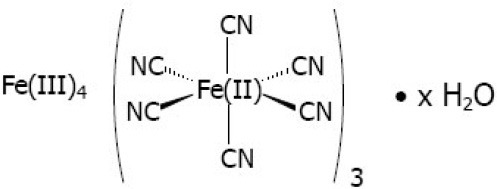	Treatment for radioactive Cesium or Thalium poisoning (approved)

### The experimental bases for a redox-mediated strategy

**Figure 2:**
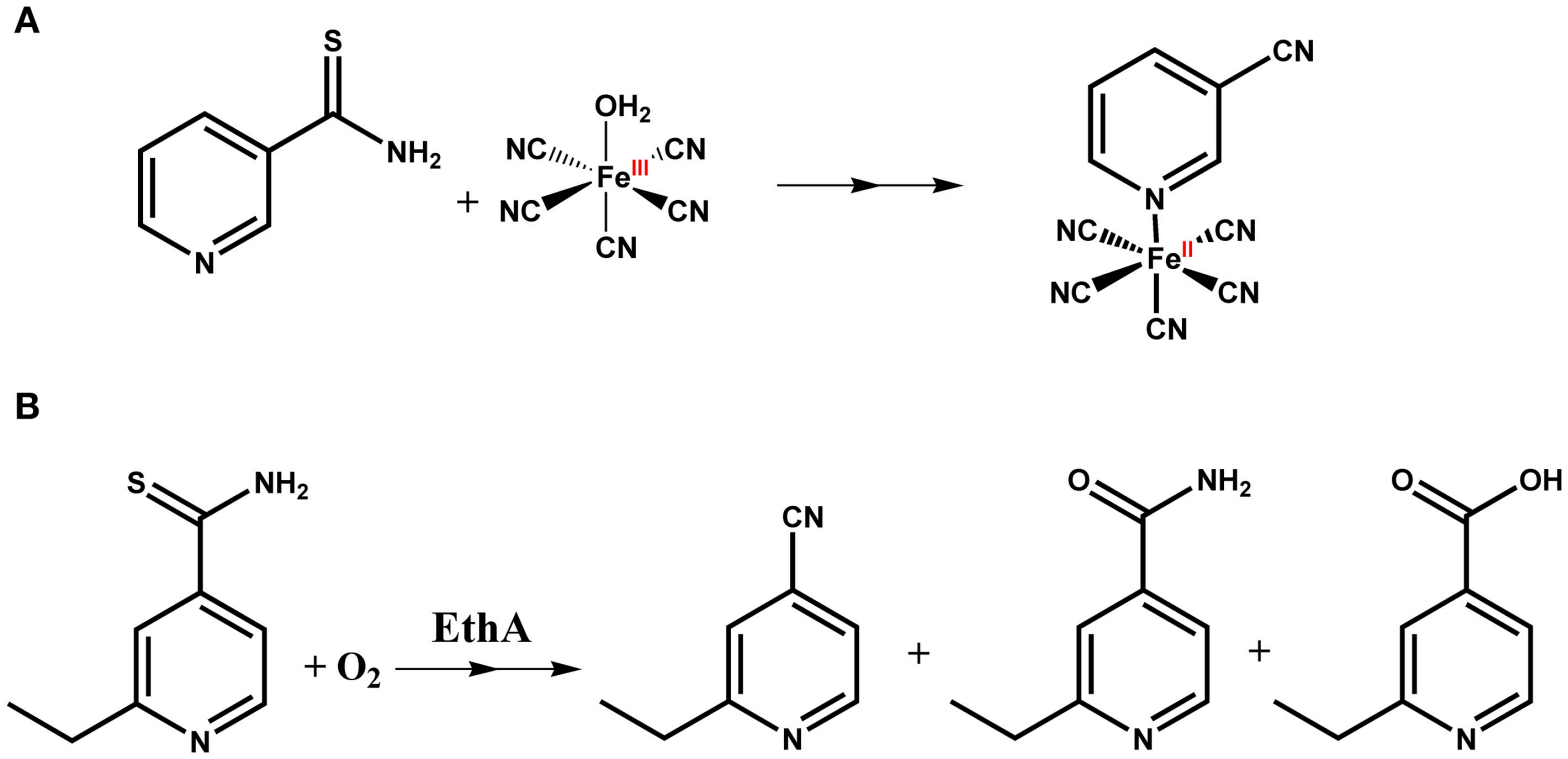
Thionicotinamide and ethionamide oxidative routes. **(A)** Reaction of thionicotinamide with [Fe^III^(CN)_5_]^2−^ and generation of reduced and nitrile-based end product. **(B)** Catalyzed reaction of oxidation of ethionamide using EthA and final metabolite products.

Early in 2000s, we proposed a redox-mediated strategy using metal complexes as an alternative route to activate anti-TB pro-drugs (e.g., isoniazid, ethionamide), which could be used against drug-resistant strains of *M. tuberculosis* (Sousa, 2000, 2003). This concept emerged from the study of thionicotinamide bound to pentacyanoferrate complexes as the thioamide has close structural similarity to the anti-TB drug ethionamide (Sousa, 2000; Sousa et al., 2005). An attempt to prepare pentacyano(thionicotinamide)ferrate(III) complex was unsuccessful as indicated by NMR and Mossbauer data of the isolated product, where an iron(II) compound was obtained instead. Further studies, showed that thionicotinamide was converted into a nitrile compound (3-cyanopyridine) bound to pentacyanoferrate(II) (IR nitrile stretching mode at 2240 cm^−1^) (Figure 2A). This phenomenon was explained as due to an electron transfer process brought about by the cyanoferrate(III) moiety binding. The oxidation of free thionicotinamide using hexacyanoferrate(III) and hydrogen peroxide yielded 3-cyanopyridine (Sousa, 2003; Laborde et al., 2016). Bioactivation of ethionamide catalyzed by flavin monooxygenase EthA and other chemical oxidizing agents also generate a nitrile product (Figure 2B) (Laborde et al., 2016, 2017).

Kinetic studies using hexacyanoferrate(III) ([Fe^III^(CN)_6_]^3−^, E_1/2_ = 450 mV vs. ENH) and aquopentacyanoferrate(III) ([Fe^III^(CN)_5_(H_2_O)]^2−^, E_1/2_ = 390 mV vs. ENH) aimed at shedding light on the mechanism of this redox reaction. [Fe^III^(CN)_6_]^3−^ is able to promote oxidation of organic molecules through one-electron mechanism of outer sphere with no bonding formed (Leal et al., 1998). On the other hand, either an outer or inner sphere mechanism could be invoked for [Fe^III^(CN)_5_(H_2_O)]^2−^, in which an organic entity can bind first to the metal replacing water then undergoing an electron transfer reaction. Interestingly, kinetic measurements of the oxidation reaction of thionicotinamide with hexacyanoferrate(III) displayed a slower rate (k_obs_ = 5.0 × 10^−7^ s^−1^) in comparison to [Fe^III^(CN)_5_(H_2_O)]^2−^ (k_obs_ = 10 s^−1^) at identical experimental conditions. An outer sphere reaction promoted by [Fe^III^(CN)_6_]^3−^ was however expected to be faster than using [Fe^III^(CN)_5_(H_2_O)]^2−^ based on their differences in driving forces, thereby favoring the former. Since the observed rates were remarkably higher for [Fe^III^(CN)_5_(H_2_O)]^2−^ (k_obs_ = 10 s^−1^), experimental pieces of evidence suggested an inner-sphere electron transfer process (Sousa et al., 2005). These results led us to propose that bound thionicotinamide as in [Fe^II^(CN)_5_(thio)]^3−^ complex, upon oxidation, would lead to fast intramolecular oxidation of the organic ligand (thio), which could not be achieved fast enough by an outer-sphere oxidation (Sousa, 2000; Sousa et al., 2005). Studies carried out with isothionicotinamide and ethionamide showed similar chemical oxidative behavior supporting that proposal (Sousa, 2003). Nevertheless, this hypothesis of a redox-mediated activation was better supported when isoniazid was used as ligand, which led to the preparation of pentacyano(isoniazid)ferrate(II) complex (IQG-607). This compound numbering was given in memory to Paul Ehrlich, father of chemotherapy, who prepared a series of arsenic-based compounds, origins of inorganic medicinal chemistry. He prepared 606 compounds of arsenic, in which the last was the most active and selective against syphilis, his “magic bullet.” We thus deemed appropriate to start counting our metal-based series from **607**. The letter code was given in honor to Dr Ícaro Moreira, PhD advisor of Dr E.H.S. Sousa at that time, where “**I**” stands for Ícaro. The QG for the four G's (from the portuguese **Q**uatro **G**) that Paul Ehrlich proposed are needed to be successful in science, from the German words: Glück, Geduld, Geschick, Geld (luck, patience, skill and money).

### Isoniazid (INH) oxidation mediated by a cyanoferrate complex

**Figure 3:**
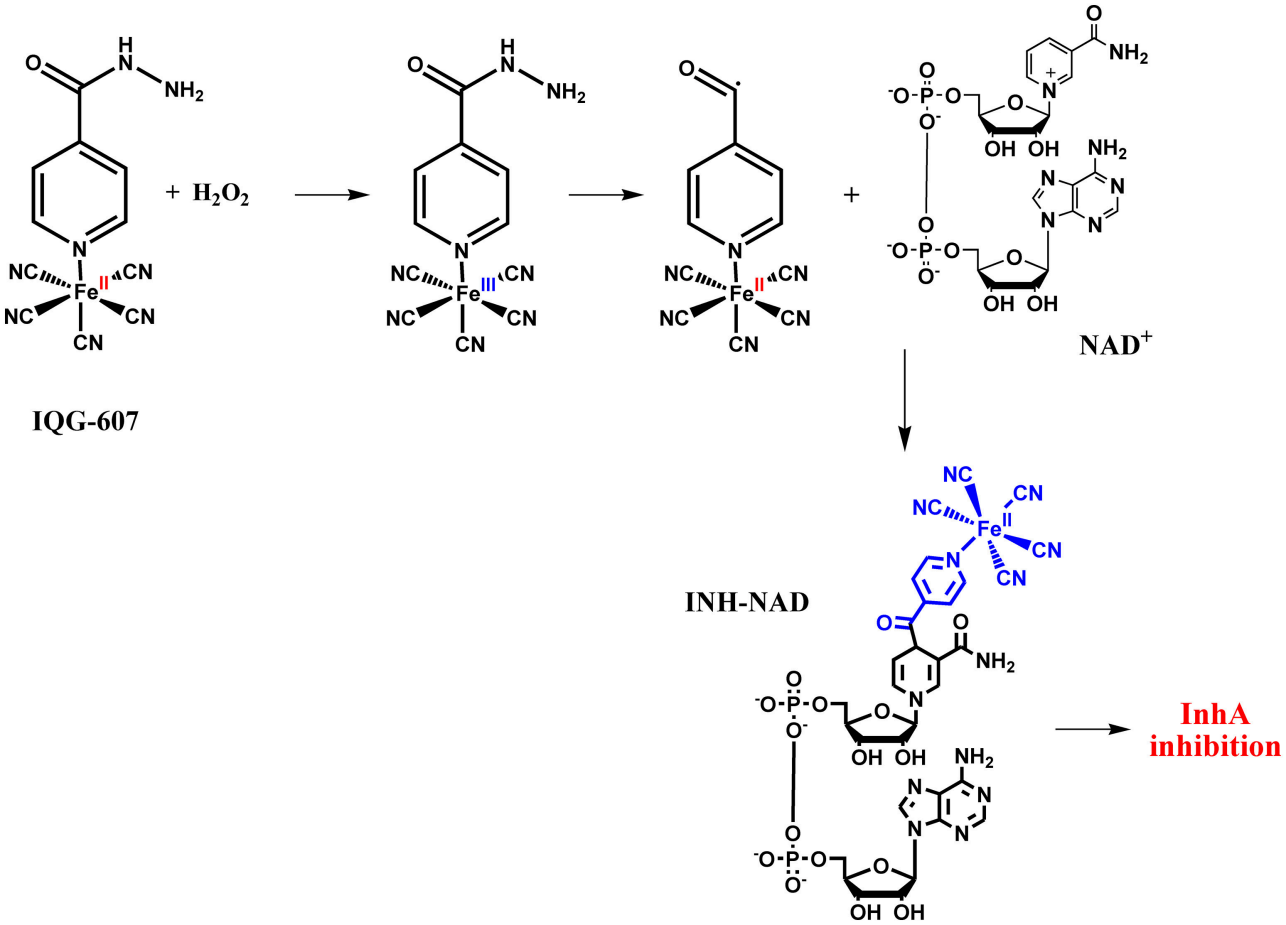
Proposed mechanisms for non-enzymatic oxidative route proposed for IQG-607 with generation of isonicotinoyl radical and likely formation of a complex adduct of INH-NAD.

INH oxidation is a slow process even using large concentrations of hydrogen peroxide for hours up to days. The *M. tuberculosis* catalase-peroxidase KatG enzyme can quickly oxidize INH, leading to its active pharmacological species. Strains of *M. tuberculosis* carrying KatG or EthA mutant proteins are resistant to, respectively, isoniazid and ethionamide. Our original proposal (Sousa, 2000, 2003) was that a metal complex containing these redox-activatable pro-drugs as ligands could work as an alternative route for mediated activation without using KatG or EthA enzymes (Figure 1). A series of *in vitro* studies were thus carried out to validate this chemical hypothesis first suggested by the work with thionicotinamide. First of all, we showed that oxidation of isoniazid bound to pentacyanoferrate(II) using hydrogen peroxide, either in excess or in stoichiometric amounts, was fast and quantitative (Sousa, 2003; Sousa et al., 2012, 2014). Actually, the oxidation of isoniazid bound to [Fe^II^(CN)_5_]^3−^ moiety seemed to be more efficient than the KatG-catalyzed chemical reaction, as it was shown that the reaction of 200 μM of isoniazid with 25 mM of H_2_O_2_ in the presence of KatG was still not complete after 6 h (Magliozzo and Marcinkeviciene, 1996). An outer sphere reaction using [Fe^III^(CN)_6_]^3−^ was carried out showing efficient oxidation of isoniazid, yielding products (isonicotinic acid and isonicotinamide) in agreement with metabolites reported for this drug (Laborde et al., 2017). Stopped-flow data showed a fast first-order reaction step when using [Fe^III^(CN)_5_(H_2_O)]^2−^ and isoniazid (k_obs_ of 12 s^−1^), which was independent of isoniazid concentration. Despite the outer sphere process of isoniazid oxidation using [Fe^III^(CN)_6_]^3−^ being efficient and pH dependent (*k* = 0.5 and 41 M^−1^ s^−1^ for pH 7.0 and 8.0, respectively), it was still at least 1000-fold slower than an inner sphere reaction using [Fe^III^(CN)_5_(H_2_O)]^2−^ (Sousa et al., 2012). These results provided support for an intramolecular oxidation reaction for a redox-mediated process of IQG-607. Since cyanoferrate(II) complexes are known to be promptly oxidized by hydrogen peroxide, which does not oxidize free isoniazid, it would be reasonable to assume that two processes occur with IQG-607: 1) Iron(II) is oxidized to iron(III), and 2) Iron(III) promotes intramolecular oxidation of isoniazid leading to oxidized ligand and reduction back to iron(II) (Figure 3). In agreement, oxidation of the isoniazid moiety of IQG-607 by superoxide ion was fast, yielding mainly isonicotinic acid as product, which does not occur with free isoniazid (Sousa et al., 2014). As analogous metabolites were observed for KatG oxidation of isoniazid, we surmised that intracellular oxidation of IQG-607 would generate the isonicotinoyl radical en route to INH-NAD adduct formation and ensuing InhA inhibition (Figure 3). Interestingly, even using stoichiometric amounts of hydrogen peroxide, α-(4-pyridyl-N-oxide)-N-tert-butylnitrone (POBN) and N-tert-butyl-α-phenylnitrone (PBN) radical traps showed strong evidence of isonicotinoyl radical production based on EPR values for their hyperfine coupling constants (POBN: a_N_ = 15.84–15.44, a_H_ = 2.7–2.9 G; PBN: a_N_ = 15.2–15.6, a_H_ = 4.5–4.9 G; for free isoniazid POBN: aN = 15.4, aH = 2.9 G, and PBN: a_N_ = 15.0–16.5, a_H_ = 3.3–3.6 G) (Sousa et al., 2014). These data showed that IQG-607 oxidation generated isonicotinoyl radical without any assistance of the KatG enzyme. Moreover, the isonicotinoyl radical was only produced if the acyl hydrazide group of bound isoniazid was present as an analogous complex containing isonicotinamide was unable to generate any isonicotinoyl radical with POBN or PBN traps upon reaction with hydrogen peroxide (Sousa et al., 2014). Some modest changes in the hyperfine coupling constants have been assigned to the isonicotinoyl radical bound to pentacyanoferrate(II) moiety. HPLC and NMR data were in agreement with the isonicotinoyl intermediate and final oxidized species bound to iron (Sousa, 2003; Sousa et al., 2014). Hydrogen NMR studies showed that the oxidized ligands (mainly isonicotinic acid) remained bound to the iron complex in the presence of hydrogen peroxide, and the metal was kept reduced with signals of diamagnetic species, low spin iron(II) (Sousa et al., 2014). Dimethylsulfoxide (DMSO) is a known competing ligand that was used in excess during HPLC experiments to displace the bound ligands after oxidative reaction. A comparison of the oxidized sample with and without addition of DMSO, along with standards for the free ligands, showed that isoniazid and derivatives are still bound to iron(II), thereby providing further evidence for the isonicotinoyl radical remaining bound to the iron of IQG-607 complex. These data also account for the slight changes in hyperfine coupling constants in comparison to free isoniazid.

*In vivo* studies (described below) showed that IQG-607 was able to abrogate mycolic acid biosynthesis, suggesting a mechanism of action analogous to isoniazid. However, only recently, we were able to show by LC-MS that IQG-607 radical could form the pentacyanoINH-NAD adduct (Laborde et al., 2018), providing evidence for its likely *in vivo* mode of action. However, genetic data have suggested otherwise (see section Elucidation of Mechanism of *M. tuberculosis* Resistance to IQG-607 below). At any rate, a redox-mediated process of a metal complex appears to be a valuable model for *in vivo* activation of other pro-drugs.

### Ruling out hydroxyl radical formation from a fenton reaction

Fenton reaction is a catalytic decomposition of H_2_O_2_ caused by iron(II) with generation of the strong oxidizing agent hydroxyl radical (OH). This radical is assumed to be able to oxidize isoniazid. As IQG-607 is an iron complex that reacts with hydrogen peroxide, it is reasonable to question whether or not a Fenton reaction is taking place and promoting isoniazid oxidation. It should be mentioned that cyanoferrate complexes are not unstable, from which release of free iron requires harsh conditions, except in a few cases (e.g., reduction of nitroprusside, [Fe(CN)_5_(NO)]^2−^; strong light exposure, extreme conditions of pH and temperature) (Kolthoff and Pearson, 1931; Bisset et al., 1981; Burger, 1985; Kuhn and Young, 2005). In agreement with low biological toxicity of these complexes, iron(III) hexacyanoferrates(II) have been used as antidotes for thallium and cesium poisoning in humans (Nielsen et al., 1990). EPR results for IQG-607 oxidation by hydrogen peroxide using 4-hydroxy-5,5-dimethyl-2-trifluoromethylpyrroline-1-oxide (FDMPO) and 5-(diethoxyphosphoryl)-5-methyl-1-pyrroline-N-oxide (DEPMPO), which are radical traps for hydroxyl or peroxyl species, showed hydroxyl radical production (Sousa et al., 2014). However, generation of the latter was also dependent on the acyl hydrazide group, suggesting that the hydroxyl radical is a product of the decomposition of this group. Oxidation of isoniazid was shown to generate hydroxyl radical without transition metal catalysis (van Zyl and van der Walt, 1994) and by a non-Fenton-type process mediated by manganese (Ito et al., 1992). Additionally, no radicals were detected for an analogous metal complex containing isonicotinamide as a ligand instead of isoniazid, which ruled out a Fenton reaction. We have recently tried to mimic IQG-607 oxidation by hydrogen peroxide using a mixture of free isoniazid and 1% of free iron(II) instead (Laborde et al., 2018). No significant oxidation of isoniazid could be detected by LC-MS for over 16 min. These results are in agreement with an intramolecular mechanism for oxidation of the isoniazid ligand of the pentacyanoferrate(II) complex.

### Analogs of IQG-607 and mechanistic insights

**Figure 4:**
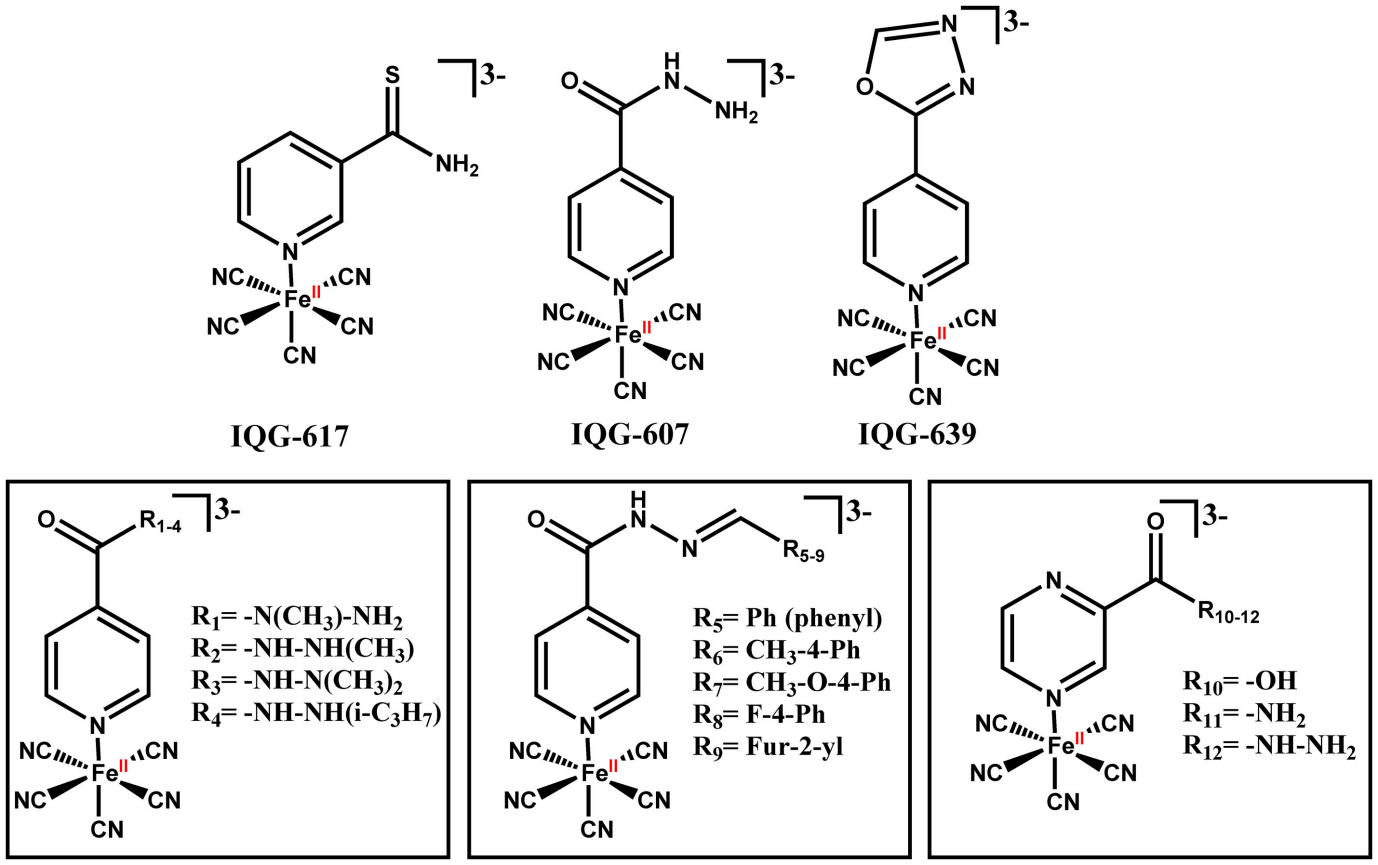
IQG-607 and other isoniazid-based iron complexes.

**Figure 5:**
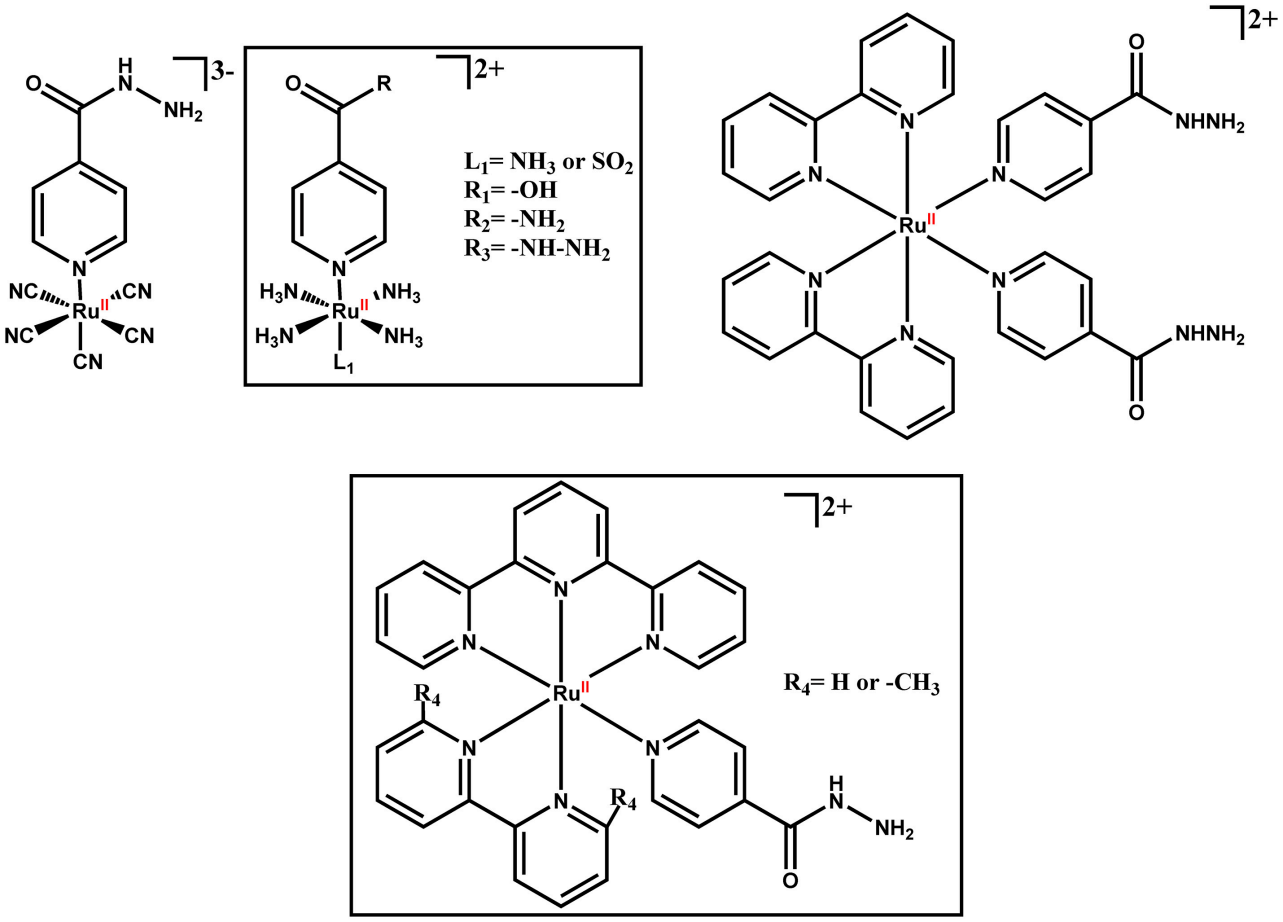
Isoniazid-based ruthenium complexes.

A number of iron- (Figure 4) and ruthenium-containing (Figure 5) analogs of IQG-607 were synthesized to provide insights into its mechanism of action. Two ruthenium complexes were prepared (Figure 5): [Ru^II^(CN)_5_(INH)]^3−^ (E_1/2_ ~993 mV vs. ENH) [Ru^II^(NH_3_)_5_(INH)]^2+^ (E_1/2_ = 356 mV vs. ENH), whose electrochemical potentials are different of IQG-607 (E_1/2_ = 549 mV vs. ENH) (Sousa, 2003; Sousa et al., 2012). Ruthenium is in the same group of iron in the periodic table and has been used to design a variety of metallodrugs with much higher stability (Barry and Sadler, 2013). Measurements of InhA enzyme inhibition were used as a proxy for their potential as anti-TB agents. Only IQG-607 inhibited wild-type and isoniazid-resistant InhA mutant protein activity (Oliveira et al., 2004). Interestingly, no KatG activation was needed and pre-incubation with NAD(H) did not speed up the inhibition process. At that time, our rationale to explain the results was that IQG-607 had a reasonably low electrochemical potential along with negative charge that would favor its interaction with InhA. The [Ru^II^(CN)_5_(INH)]^3−^ was likely unable to inhibit InhA due to its high electrochemical potential as it is fairly analogous to IQG-607 even in size. On the other hand, [Ru^II^(NH_3_)_5_(INH)]^2+^ was also unable to inhibit InhA despite having an electrochemical potential smaller than IQG-607, and its positive charge could be invoked to impair protein binding. Surprisingly, [Ru^II^(NH_3_)_5_(INH)]^2+^ was able to inhibit *M. tuberculosis* growth with an MIC value of 1.56 μg/mL (Sousa, 2003) by a not yet known mechanism of action. Others reported antitubercular activity for ammines of Ru(II) isoniazid complexes with good MIC values (approximately 0.88 μg/mL), and a theoretical correlation between anti-TB activity and stability of isonicotinoyl radical, not the electrochemical potential of the metal center, was proposed (Aguiar et al., 2015). Other ruthenium-based complexes with and without isoniazid bound have been reported to exhibit anti-TB activity (Pavan et al., 2011; Garner et al., 2017; Silva et al., 2017; Smith et al., 2017).

A series of cyanoferrate(II) complexes containing isonicotinoylhydrazone ligands (Figure 4) was prepared and measurements of InhA enzyme inhibition and antitubercular activity against a drug-susceptible *M. tuberculosis* strain were carried out (Gazzi et al., 2017). There was a modest difference in electrochemical potential among all of those complexes, ranging from 337 to 426 mV vs. Ag|AgCl, which is expected as they are bound through an analogous pyridinic nitrogen. On the other hand, these compounds showed significant structural differences that could aid their interaction with InhA enzyme. Interestingly, we found that some of these complexes were better *in vitro* inhibitors of InhA than IQG-607 with inhibition rate values 10-fold larger, though with similar MIC values (Gazzi et al., 2017).

An oxadiazole derivative of isoniazid bound to pentacyanoferrate(II), named IQG-639 (sodium pentacyano[2-metil-5-(piridin-4-il)-1,3,4-oxadiazole]ferrate(II) (Figure 4), inhibited InhA enzyme activity (t_1/2_ = 1.37 min) (Sales, 2008; Rodrigues-Junior et al., 2012). This compound also inhibited the growth of *M. tuberculosis* pan-sensitive and isoniazid-resistant clinical strains (MIC for WT = 0.5 μg/mL; *inhA* structural gene mutant (S94A) = 2.0 μg/mL; *inhA* operon promoter region mutant [C(−15)T] = 4.0 μg/mL) (Rodrigues-Junior et al., 2012). However, this compound showed no activity in a murine model of TB infection (Rodrigues-Junior et al., 2012).

Recent efforts made in collaboration with Dr Bernardes-Genisson have focused on the modification of the proximal and distal nitrogen of the acyl hydrazide group of isoniazid bound to pentacyanoferrate(II) complex (Laborde et al., 2018). These metal complexes could promote oxidation of isoniazid despite harboring chemical modifications on the nitrogen of the hydrazide group. All modifications disrupted the production of aroyl radical and led to alkyl radical production instead, suggesting these complexes would not be able to lead to INH-NAD adduct formation. In agreement with the latter, these complexes showed no *in vitro* growth inhibition of *M. tuberculosis* (Laborde et al., 2018).

### Chemical properties of IQG-607

The IQG-607 complex was prepared by mixing isoniazid in excess with aminpentacyanoferrate(II), in water under anaerobic and light protected conditions, from which an orange solid was isolated containing a pyridine nitrogen bound complex of sodium pentacyano(isoniazid)ferrate(II). This isolated complex is an oxygen- and light-sensitive compound, which is highly soluble in water (>40 mM). A pilot scale preparation has been carried out with production of several grams of this complex aiming at, hopefully, future clinical trials (Basso et al., 2010). The cyanide stretching mode of infrared measurements can differentiate between cyanoferrates of iron(II) and iron(III). Unfortunately, most of the vibrational modes of the organic ligands are of low intensity or even not seen. For IQG-607, we observe a large band with maximum at 2,044 cm^−1^ and Fe-C at 563 cm^−1^, in agreement with iron(II). Electronic spectrum exhibits a well-defined band in the visible range with maximum at 436 nm and absorptivity of 4.0 × 10^3^ mol^−1^ L cm^−1^. This band can be used for quality purposes to monitor its degradation either by light, oxygen or competition with other ligands, whose products usually show a band shift toward 415 nm. A pentacyanoferrate(II) complex can promote electron delocalization toward the ligand, a phenomenon called back bonding effect. This process can alter the electronic distribution of the bound ligand and might affect its own reactivity, which can be indicated by NMR shifts of hydrogen of INH. We have observed a significant downfield shift on NMR signal for the meta-hydrogens of isoniazid (Δ = 0.26 ppm), supporting backbonding effect, which might influence oxidation of bound ligand. Despite these properties, an analogous complex containing isonicotinamide shows almost identical characteristics, which make difficult to differentiate them. Fortunately, the major product of IQG-607 oxidation is the isonicotinic acid, whose complex with pentacyanoferrate shows well distinct electronic spectra, while the electrochemical potential is fairly similar. More recently, a mass spectrometry method was developed to identify the main ions along with decomposition products (Laborde et al., 2018), which will be useful for *in vivo* metabolism studies.

## InhA inhibition by IQG-607

### Activity of IQG-607 against WT and I21V InhA enzymes

IQG-607 was shown to inhibit WT and isoniazid-resistant I21V InhA mutant enzymes in a time-dependent manner (Oliveira et al., 2004). Inactivation of WT InhA enzyme occurs faster in the absence of NADH, as indicated by the apparent first-order rate constant values obtained in the absence of NADH [327 (± 34) × 10^−3^ min^−1^, t_1/2_ = 2.1 ± 0.2 min], in the presence of 10 μM of NADH [65 (± 4) × 10^−3^ min^−1^, t_1/2_ = 10.7 ± 0.7 min), and in the presence of 100 μM of NADH (15.7 (± 0.7) × 10^−3^ min^−1^, t_1/2_ = 44 ± 2 min] (Oliveira et al., 2004). These data suggest that the mechanism of action of IQG-607 involves an interaction with the NADH binding site of InhA, since the presence of NADH protects WT InhA from inactivation by IQG-607 as indicated by the apparent first-order constants (Oliveira et al., 2004). Computational docking of IQG-607 to InhA:NADH binary complex indicated that this compound and the extended conformation of NADH cannot simultaneously occupy the cofactor binding site of InhA crystallographic structure (Oliveira et al., 2006). Molecular docking data also showed that IQG-607 preferentially occupies the pyrophosphate and nicotinamide sites in the NAD(H) binding pocket (Oliveira et al., 2006), which corroborates with the lower apparent first-order rate constant values for InhA inactivation in the presence of NADH. On the other hand, isoniazid derivatives produced by KatG oxidative reaction only inhibited WT InhA activity in the presence of NADH, with an apparent first-order constant value of 8.9 (± 0.2) × 10^−3^ min^−1^ (Basso et al., 1996). Time-dependent experiments were performed to evaluate if the 2-*trans*-dodecenoyl-CoA (DD-CoA) substrate protects against InhA inactivation by IQG-607 (Oliveira et al., 2006). The apparent first-order rate constant values for InhA inactivation decreased as a function of increasing DD-CoA concentration, which suggests the inhibition mechanism of IQG-607 involves interaction with both NADH and DD-CoA binding sites, suggesting a competitive mode of inhibition with respect to both substrates (Oliveira et al., 2006). Moreover, IQG-607 was shown to inhibit the activity of I21V InhA mutant protein with similar apparent first-order rate constant obtained for WT enzyme, as indicated by the values observed in the absence of NADH [315 (± 38) × 10^−3^ min^−1^, t_1/2_ = 2.2 ± 0.3 min] (Oliveira et al., 2004). On the other hand, inactivation of I21V and I95P InhA mutants by KatG-produced INH-NAD adduct resulted in decreased apparent first-order rate constant values of 3.3 (± 0.20) × 10^−3^ min^−1^ and 0.34 (± 0.09) × 10^−3^ min^−1^, respectively, when compared with WT enzyme [4.48 (± 0.17) × 10^−3^ min^−1^] (Basso et al., 1998). Interestingly, pre-incubation studies of IQG-607 and NADH indicated that there is no slow formation of an intermediate compound capable of inhibiting WT and I21V InhA enzymes (Oliveira et al., 2004). These data indicated that IQG-607 is able to inactivate InhA harboring I21V found in isoniazid-resistant clinical isolates (Basso et al., 1998; Oliveira et al., 2004), requires no activation by KatG and does not depend on NADH for enzymatic inhibition.

### Slow-binding inhibition kinetics of InhA by IQG-607

Slow-binding inhibition experiments were performed to elucidate the kinetic mechanism of time-dependent inhibition of InhA by IQG-607 (Oliveira et al., 2006). The pattern of progress curves obtained over time in the presence of different concentrations of IQG-607 showed that larger concentrations of IQG-607 caused the steady state to be reached faster but resulted in a lower steady-state velocity (Oliveira et al., 2006). The progress curves indicated that IQG-607 is a slow-binding inhibitor of WT InhA. Slow-binding inhibitors establish the equilibrium among enzyme, inhibitor and enzyme-inhibitor complexes slowly on the steady-state time of seconds to minutes (Morrison and Walsh, 1988). The mechanism of action B, described by Morrison and Walsh (1988), involves the rapid formation of an initial complex (EI), which undergoes a slow isomerization reaction (conformational change) to EI^*^, where the inhibitor is more tightly bound to enzyme (Oliveira et al., 2006). This mechanism of inhibition presents an overall dissociation constant (K_i_^*^) lower than the dissociation constant for initially formed complex EI (K_i_), in which k_6_ is < k_5_ [K_i_^*^ = K_i_ k_6_/(k_5_ + k_6_)] and when k_5_ and k_6_ are slower than all other steps (Morrison and Walsh, 1988). Values of 32 ± 3 μM and 0.41 ± 0.01 min^−1^ were obtained for the rapidly reversible dissociation constant for WT InhA-IQG-607 binary complex (K_i_) and for the forward rate constant (k_5_), respectively, when plotting apparent pseudo-first order rate constant (k_obs_) as a function of IQG-607 concentration, which displayed a hyperbolic increase (Oliveira et al., 2006). These data are consistent with the mechanism B of inhibition of InhA by IQG-607, and ruled out the other two basic mechanisms that describe reversible slow-binding inhibition of enzyme-catalyzed reactions (Oliveira et al., 2006). The INH-NAD adduct was also shown to be a slow, tight-binding competitive inhibitor (Rawat et al., 2003), showing a mechanism of InhA inhibition similar to IQG-607. The plots of k_obs_ values vs. IQG-607 concentrations for the I21V, I47T, and S94A InhA mutant enzymes displayed the same mechanism of inhibition described for WT InhA (Vasconcelos et al., 2008). Moreover, the values of the forward rate constants for EI^*^ formation by IQG-607 complex with WT, I47T, and I21V were, respectively, 3.2, 4.4, and 3.8 fold larger than the value determined for activated isoniazid (Rawat et al., 2003; Vasconcelos et al., 2008).

### Dissociation of IQG-607 from InhA and overall inhibition constant (K_i_^*^)

The reverse rate constant for the conversion of WT InhA-inhibitor complex EI^*^ to EI was evaluated to demonstrate reversible binding and to obtain a reliable estimate for *k*_6_-value (Oliveira et al., 2006). Values for k_6_ of 1.1 (± 0.1) × 10^−3^ min^−1^, 1.18 (± 0.09) × 10^−3^ min^−1^, and 1.01 (± 0.03) × 10^−3^ min^−1^ were obtained for, respectively, 25, 75, and 125 μM of IQG-607 (Oliveira et al., 2006). The similar k_6_ values found with different inhibitor concentrations indicated that IQG-607 rebinding was not significant, and that the true reverse isomerization constant could be estimated (Oliveira et al., 2006). Moreover, the half-time of the limiting step for inhibitor dissociation from E^*^-IQG-607 binary complex was estimated to be 630 ± 28 min (Oliveira et al., 2006). This slow rate of dissociation may enhance inhibitor's effectiveness. The k_5_/k_6_ ratio of the isomerization process of E-IQG-607 to E^*^-IQG-607 was estimated as 0.41 min^−1^/1.1 × 10^−3^ min^−1^ (Oliveira et al., 2006). The k_5_/k_6_ ratio indicates the index of the accumulation of EI^*^ and the energetics of its formation, which means the higher the value of k_5_/k_6_ ratio, the longer lived is EI^*^ and more likely inhibitor is to have a desirable *in vivo* lifetime (Oliveira et al., 2006). The overall dissociation constant (K_i_^*^) was estimated to be 70 ± 10 nM, which is a function of the relative magnitudes of the forward (k_5_) and reverse (k_6_) rates for InhA-IQG-607 isomerization process (Oliveira et al., 2006). A comparison between the K_i_ value (32 μM), which is the inhibitor dissociation constant value for the rapidly reversible EI complex formation, and the K_i_^*^ (70 nM) indicates the E^*^-IQG-607 is more stable than the initially formed E-IQG-607 complex (Oliveira et al., 2006). Interestingly, a dissociation rate constant value of 0.13 min^−1^ (t_1/2_ = 5.3 min) was estimated for INH-NAD adduct from InhA (Rawat et al., 2003), suggesting a larger residence time for IQG-607 in comparison to INH-NAD.

Dissociation constant of IQG-607 from InhA and overall inhibition constant were also determined for I47T, I21V, and S94A mutants (Vasconcelos et al., 2008). The k_6_ values for I47T, I21V, and S94A InhA and IQG-607 binary complexes were determined to be, respectively, 1.2 (± 0.1) × 10^−3^ min ^−1^, 1.27 (± 0.2) × 10^−3^ min ^−1^ and 1.45 (± 0.2) × 10^−3^ min ^−1^ (Vasconcelos et al., 2008). The half-time for inhibitor dissociation from E^*^-IQG-607 binary complexes were 578, 546 and 478 min for, respectively, I47T, I21V, and S94A, which are similar to the value obtained for WT InhA (630 min) (Vasconcelos et al., 2008). Moreover, The K_i_^*^ values for WT, S94A, I21V, and I47T InhA enzymes were, respectively, 225, 205, 263, and 303 nM, indicating that the amino acid substitutions in the mutant enzymes did not affect IQG-607 affinity for InhA (Vasconcelos et al., 2008). These data suggested that IQG-607 might be able to inhibit INH-resistant clinical isolates harboring mutations in the *inhA* structural gene.

### Two-step mechanism for WT InhA inhibition by IQG-607

To provide further support for the mode of inhibition, two-step inactivation experiment of WT InhA by IQG-607 was performed by determining the values for apparent first-order rate constant of inactivation (k_inact_) from a plot of residual enzyme activity vs. time for each inhibitor concentration (Oliveira et al., 2006). The k_inact_ values were plotted against IQG-607 concentration in a preincubation mixture, and values of 0.51 ± 0.03 min^−1^ and 73 ± 14 μM were obtained for k_5_ (forward isomerization rate constant), and K_i_ (dissociation constant for formation of EI), respectively (Oliveira et al., 2006). These values are in agreement with the ones calculated in slow-binding inhibition experiments, which lend support to the two-step mechanism for WT InhA inactivation by IQG-607 (Oliveira et al., 2006).

In summary, enzymatic inhibition and computational docking studies demonstrated that IQG-607 inhibits the WT InhA and the I21V, S94A, and I47T mutant enzymes preferentially in the absence of NAD or NADH, and without requiring KatG catalase-peroxidase activation. These results suggested that IQG-607 is not a pro-drug as isoniazid, since it both interacts with the NADH binding site of InhA and does not require formation of a chemical complex with NAD(H) to inhibit InhA enzyme activity. As these findings could not be reconciled with the self-activation mechanism followed by adduct formation and InhA inhibition (described above in detail: sections The Experimental Bases for a Redox-Mediated Strategy, Isoniazid (INH) Oxidation Mediated by a Cyanoferrate Complex, and Analogues of IQG-607 and Mechanistic Insights), further efforts were pursued to try to elucidate the mechanism of action and resistance of *M. tuberculosis* to IQG-607 by employing a genetic approach as described in section Elucidation of Mechanism of *M. tuberculosis* Resistance to IQG-607 below.

## *M. tuberculosis* growth inhibition studies

The minimum inhibitory concentration (MIC) for IQG-607 growth inhibition of *M. tuberculosis* H37Rv reference strain, at an early stage of our efforts, was determined by the radiometric BACTEC system and a value of 0.2 μg/mL was obtained, which was comparable to the MIC value for INH (0.02–0.2 μg/mL) (Oliveira et al., 2004). The MIC determined by the colorimetric microplate resazurin-based Alamar Blue assay (MABA) assay was 0.25 μg/mL (Basso et al., 2010). MIC values of 1.0 μg/mL for S94A and 4.0 μg/mL for [C(-15)T] INH-resistant mutants were also reported for IQG-607 (Basso et al., 2010). The MIC values for these two drug-resistant strains were larger than 16 μg/mL for INH (Silva et al., 2003). However, the two clinical isolates available at that moment and used for these experiments did not have their genome completely sequenced and other mutations/mechanisms of resistance could thus exist.

To try to determine the mechanism of action of IQG-607, the effects of this compound were analyzed on mycolic acid synthesis by *in vivo* radiolabelling, extraction and analysis of lipids of *M. tuberculosis* cells. IQG-607 totally blocked the synthesis of mycolic acids, as indicated by incorporation of the radiolabelled precursor acetate (Rodrigues-Junior et al., 2014). Moreover, IQG-607 did not inhibit the synthesis of non-hydroxylated fatty acids, similar to INH (Rodrigues-Junior et al., 2014). These observations were in accordance with prior suggestions based on *in vitro* enzymatic inhibition results (Oliveira et al., 2004, 2006; Vasconcelos et al., 2008).

*Mycobacterium tuberculosis*-infected macrophages were employed to evaluate whether or not IQG-607 had any intracellular activity. A broad range of concentrations of either INH or IQG-607, varying from 0.5 to 360 μM, resulted in a significant bacterial load reduction for all concentrations. Importantly, IQG-607 (360 μM) produced a better reduction in Colony Forming Units (CFU) counts as compared to INH at the same concentration (Rodrigues-Junior et al., 2014). Additionally, IQG-607 lowered the CFU counts with percentages of reduction statistically similar to those observed for rifampicin (RIF) (Rodrigues-Junior et al., 2014). These results suggest that IQG-607 can cross the macrophage membrane and reach the bacilli, killing them within the phagosome.

The activity of IQG-607 was also determined in *M. tuberculosis*-infected mice (Rodrigues-Junior et al., 2012). Firstly, the murine infection model was validated after an intravenous injection of a suspension containing *M. tuberculosis* cells in mice: splenomegaly and lung tissue damage were observed in the infected animals. Moreover, the morphological features of the granuloma formation were found in lungs of infected mice (Rodrigues-Junior et al., 2012). According to macroscopic evaluation, either the treatment with the reference drug INH or the test compound IQG-607 effectively ameliorated the lung aspect, and reduced the spleen weights, when comparing to untreated controls. CFU counts showed that the bacterial loads in the lungs and spleens from IQG-607-treated mice were markedly lower than those from untreated controls, after 4 and 8 weeks of treatment (Rodrigues-Junior et al., 2012). Based on these findings, it is tempting to suggest that IQG-607 is absorbed after oral administration, reaching the lungs and being able to reach the bacilli, and killing them within the phagosome of macrophages. The dose of IQG-607 used for treating the infected mice for 28 and 56 days, 250 mg/kg, was chosen according to the acute oral toxicity assays, as either 250 or 500 mg/kg did not cause any toxic clinical signs or mortality in male and female mice, showing favorable toxicological features (Basso et al., 2010).

It was evaluated whether IQG-607 activity followed a dose-response pattern. No significant reduction in spleen or lung CFU loads or any lowering in the spleen weights were observed after 28 days of treatment with 5 mg/kg of IQG-607. Using mice as an animal model, 10 mg/kg of IQG-607 was the lowest dose that displayed a significant activity. The effects of IQG-607 as a bacteriostatic or a bactericidal molecule were also investigated. Treatment with IQG-607 significantly reduced the bacterial loads from lungs and spleens compared with both early and late control groups. As INH, IQG-607 displayed bactericidal activity in this TB model (Rodrigues-Junior et al., 2012). Early control group represented those animals that were euthanized just at treatment onset (and did not receive any treatment), while late control group represented those mice that remained receiving vehicle during the treatment period and were euthanized at the same day as treated mice.

Based on these data, it was proposed that IQG-607 had an MIC value similar to that reported to INH, exhibiting a favorable intracellular activity, and satisfactory *in vivo* efficacy, being absorbed after oral route of administration, lacking any apparent toxicity in mice. This scenario prompted us to further investigating the safety/toxicity of IQG-607.

## Toxicity studies

When evaluating a new drug candidate, it is mandatory to examine the toxicological properties and safety profile of the new molecule, by using both *in vitro* and *in vivo* approaches, in an attempt to guarantee its safety in humans. *In vitro* evaluations are complementary to *in vivo* tests (Andrade et al., 2016). The cytotoxicity of IQG-607 was thus assessed against macrophages derived from peripheral blood mononuclear cells, and against three additional cell lineages: Vero, HaCat, and HepG2 (Amorim et al., 2017). Incubations (for 72 h) with varying concentrations of IQG-607 up to 2,000 μM did not significantly affect cell viability, while treatments with higher concentrations (4, 8 or 16 mM) reduced the cell viability to percentages lower than 50 % for the three lineages evaluated. It was estimated that the IC_50_ value was larger than 2,000 μM for the eukaryotic cell lines tested (Amorim et al., 2017). Comparatively, the IC_50_ value for INH was determined to be approximately 1,100 μM after incubation for 72 h in cultured human hepatocytes (Shen et al., 2008), or approximately 1,500 μM after incubation for 48 h in HepG2 cells (Anju et al., 2016), both assessed by the MTT method. The selectivity index (SI) for IQG-607 was estimated (SI = IC_50_/MIC) to be higher than 4,000, considering the value of MIC = 0.25 μg/ml, which corresponds to 0.5 μM (Basso et al., 2010) and IC_50_ of 2,000 μM (Amorim et al., 2017). Accordingly, as suggested by the Tuberculosis Antimicrobial Acquisition & Coordinating Facility of USA, for a compound to move forward through screening programs, its SI values should be >10. The SI value for IQG-607 (SI ≥ 4,000) thus warranted further efforts to be pursued in *in vivo* toxicological studies. Incidentally, the SI value for INH can be estimated to be larger than 6,000, considering the value of MIC = 0.025 μg/ml, which corresponds to 0.18 μM (Rastogi et al., 1996). Noteworthy, IQG-607 did not exhibit genotoxic effects even when IQG-607 was incubated at high concentration (1 mM), as revealed by the alkaline comet assay, and it did not induce DNA damage in HepG2 cells (Amorim et al., 2017). The mutagenic potential of IQG-607 is currently being tested by using different strains of *Salmonella typhimurium* (Ames test, unpublished data).

Additional studies to evaluate IQG-607 toxicological and safety parameters in rodents were performed. The toxicological findings after single and 90-days repeated oral dosing of IQG-607 in Wistar Hannover rats were promising (Rodrigues-Junior et al., 2017b). A single oral dosing of IQG-607 (300 or 2,000 mg/kg) to female rats did not cause any mortality, even when the high dose of 2,000 mg/kg was administered. Thus, the oral median lethal dose (LD_50_) value for IQG-607 was assumed to be higher than 2,000 mg/kg for female rats (Rodrigues-Junior et al., 2017b). Comparatively, the LD_50_ for rats orally dosed with INH is 1,250 mg/kg (RTECS, 1979). For repeated-dose toxicity studies, three different doses were tested (25, 100, and 300 mg/kg) and the highest dose was chosen with the aim to induce toxicity, but not severe suffering, according to recommendations from OECD (1998). The repeated oral administration of 300 mg/kg of IQG-607 caused 20% of deaths, in rats of both sexes (Rodrigues-Junior et al., 2017b). The main clinical signs and toxic reactions observed after single and repeated administration of IQG-607 to rats (Rodrigues-Junior et al., 2017b) are summarized in Table 2. Importantly, parameters related to hepatic damage (aspartate and alanine transaminase, alkaline phosphatase, bilirubin, total protein, and histopatholoy) were found not altered for IQG-607 even after the administration of the highest dose tested, 300 mg/kg, in the repeated 90-day schedule, for both male and female rats (Rodrigues-Junior et al., 2017b). INH, in turn, caused significant hepatic damage, when lower doses were given to rats for shorter periods of administration (50 mg/kg for 21 days, and 25 mg/kg for 45 days) (Ergul et al., 2010; Raghu and Karthikeyan, 2016). Additionally, the oral LD_50_ for IQG-607 in mice, 1,000 mg/kg (Basso et al., 2010), is larger than the value reported for INH, 133 mg/kg (RTECS, 1979), further reinforcing the favorable safety profile for IQG-607 in comparison to INH.

**Table 2:** Main findings and clinical observations recorded after single or repeated administration of IQG-607 in rats (Rodrigues-Junior et al., 2017b).

**Single dosing (300 or 2,000 mg/kg)**	**Repeated-dose oral toxicity test (75, 150, or 300 mg/kg)**	
	**Male**	**Female**
No mortality	Mortality (300 mg/kg, 2/10)	Mortality (300 mg/kg, 2/10)
No important loss of weight	No significant body weight alteration	No significant body weight alteration
No changes in food intake or water consumption	No changes in food intake or water consumption	No changes in food intake or water consumption
No gross lesions at necropsy	Excessive salivation (300 mg/kg, 9/9)	Excessive salivation (300 mg/kg, 9/9)
Breathing difficulties: 300 mg/kg, 1/6; 2,000 mg/kg, 1/6	Blood around nose and eyes (1/9)^*^	Blood around eyes (1/10)^*^
Diarrhea (300 mg/kg, 1/6)	No pupillary response (1/9)^*^	No pupillary response (1/10)^*^
Pupillary contraction/dilatation: 300 mg/kg, 1/6; 2,000 mg/kg, 2/6	Piloerection (1/9)^*^	Skin and fur alterations (1/10)^*^
Cachexia (300 mg/kg, 1/6)	Tremors (1/9)^*^	Difficulty for breathing (1/10)^*^
Blood around nose and eyes (2,000 mg/kg, 3/6)	Reduction on total cholesterol levels (100 or 300 mg/kg)	Piloerection (1/10)^*^
Persistent alteration in skin (2,000 mg/kg, 1/6)	Reduction on triglyceride levels (100 mg/kg)	Ptosis (1/10)^*^
	Increased relative weight of lungs (300 mg/kg)	Lethargy, lordosis, and tremors (1/10)^*^
		Increased relative weight of lungs (300 mg/kg)

* *Signs with low incidence which were also observed in animals that received only vehicle, and are not likely to be related to IQG-607 treatment or dose*.

Taking into consideration that toxicological investigations in a non-rodent species are mandatory and that they could predict safety, tolerability and potential adverse and harmful reactions of a drug in humans, IQG-607 was also tested in minipigs, following single and repeated schedules of administration, as recommended by OECD (OECD, 2001; Rodrigues-Junior et al., 2017a). Of importance, minipigs are considered close to humans in terms of anatomy, biochemistry and physiology (Bode et al., 2010). Therefore, testing in minipigs is considered predictive of human reactions, bringing a more accurate estimate of harmful effects to humans. A single dose of IQG-607 (220 mg/kg) administered to both male and female minipigs did not cause mortality, morbidity, body weight changes, alteration of food and water consumption, or alterations at necropsy (Rodrigues-Junior et al., 2017a). It is important to mention that the dose of 220 mg/kg corresponds to the dose of 1,000 mg/kg, for rodents, as calculated based on the body surface area. The LD_50_ value for IQG-607 is then assumed to be larger than 220 mg/kg in minipigs. Comparatively, the LD_50_ for INH is 50 and 325 mg/kg, for other mammal species, dogs and cats, respectively (RTECS, 1979). For the repeated-dose experiments in minipigs, the administered doses (15, 30, and 65 mg/kg) corresponded to those used in the repeated administration study in rats (Rodrigues-Junior et al., 2017b). Interestingly, the repeated oral dosing of IQG-607 to minipigs did not cause deaths (Rodrigues-Junior et al., 2017a), contrary to the observations in rats (Rodrigues-Junior et al., 2017b). However, in the sub-chronic study, mild adverse effects were observed (Rodrigues-Junior et al., 2017a). These data are summarized in Table 3. The histopathological alterations of the organs from IQG-607-treated groups were also observed in minipigs from the untreated group, indicating that those observations were not caused by IGQ-607 treatment (Rodrigues-Junior et al., 2017a).

**Table 3:** Main clinical findings observed after single or repeated administration of IQG-607 in minipigs (Rodrigues-Junior et al., 2017a).

**Single dose (220 mg/kg)**	**Repeated-dose oral toxicity test**		
	**15 mg/kg**	**30 mg/kg**	**65 mg/kg**
No mortality	No mortality	No mortality	No mortality
No body weight loss		Body weight increase less than controls	Body weight increase less than controls
No alteration in the pattern of food or water consumption	No significant difference in the final mean of body weights	No significant difference in the final mean of body weights	No significant difference in the final mean of body weights
No gross alterations at necropsy	Diarrhea (3/8), no aberrant gastrointestinal histopathological changes	Diarrhea (3/8), no aberrant gastrointestinal histopathological changes	Diarrhea (3/8), no aberrant gastrointestinal histopathological changes
Apathy (1/8)	Alopecia and hair loss	Alopecia and hair loss	Alopecia and hair loss
Alopecia (1/8)	Increased creatinine levels	Increased glucose levels	Increased cholesterol levels
Diarrhea (1/8), likely related to the dose	Increased prothrombin time		Decrease globulin levels
Vomiting (4/8)			

The findings regarding toxicity investigation protocols have shown an overall favorable safety profile for IQG-607 in cells, mice, rats, and minipigs (Basso et al., 2010; Amorim et al., 2017; Rodrigues-Junior et al., 2017a,b). However, comparative toxicological studies are currently underway to investigate whether or not IQG-607 could be employed as an alternative to INH as the latter may cause may cause toxic side effects (Kass and Shandera, 2010; Wang et al., 2016) such as drug-induced liver injuries (Saukkonen et al., 2006).

## Methods for quantification and pharmacokinetic studies

Initially, a method of adsorptive stripping voltammetry and application of a differential pulse scan in the stripping step (DPAdSV) was developed to detect and quantify IQG-607 (Dadda et al., 2015). This method was employed to monitor IQG-607 chemical stability after 30 days of storage at 4 and 37°C (protected from light) (Dadda et al., 2015). These experiments were performed concomitantly with enzyme inhibition assays (Oliveira et al., 2006). The results showed that IQG-607 is stable for at least 1 week at 4°C in solution as no significant changes were observed for both the electrochemical behavior and InhA enzyme inhibition rate constant. After 1 week of storage at 37°C, changes in the electrochemical behavior with concomitant loss of InhA enzyme inhibitory activity were observed. These results indicate that intact IQG-607 is the inhibitor of InhA enzyme activity, and not its degradation products generated under storage conditions (isonicotinic acid and possibly others that need to be investigated) (Dadda et al., 2015).

More recently, a high performance liquid chromatographic (HPLC) method was developed and validated for quantification of IQG-607 in mouse plasma (Dadda et al., 2018). This analytical method was employed in a pharmacokinetic study in mice after intravenous (i.v.) and oral (fasted and fed conditions) administration of IQG-607. The amounts of IQG-607 in plasma were quantified at various time points for up to 2.5 h, and non-compartmental analysis of the data was performed to determine the pharmacokinetic parameters. After i.v. administration of IQG-607 (50 mg/kg) in mice, the compound showed a short half life (*t*_1/2_ = 1.1 h), a high clearance (*CL* = 3.9 L/h/kg) and a moderate volume of distribution at steady state (Vdss = 1.2 L/kg). The compound also presented the following pharmacokinetic parameters: elimination rate constant (Ke) = 0.61 h^−1^, area under the concentration-time curve from 0 to time *t* (AUC_0 → *t*_) = 12.84 μg/L h, area under the plasma concentration–time curve from time zero to infinity (AUC_0 → ∞_) = 13.69 μg/L h and a mean residence time (MRT) = 0.31 h (Dadda et al., 2018). After oral administration of 250 mg/kg to fasted and fed mice, the peak concentration of IQG-607 in plasma (*C*_max_) (1.23 and 1.72 μg/mL, respectively) was achieved after 0.25 h (*t*_max_). The AUC_0 → *t*_ and AUC_0 → ∞_ were 1.7 and 2.6 μg/L h, respectively, for fasted group, while for the fed group the values were 2.0 and 2.7 μg/L h, respectively. Under fasting condition, the Ke and *t*_1/2_ elimination values were, respectively, 0.43 h^−1^ and 1.59 h with similar values under fed condition (Ke = 0.55 h^−1^ and *t*_1/2_ = 1.3 h). The *CL* was similar for both fasted and fed conditions (3.6 L/h/kg). The MRT was also similar in the fasted (2.3 h) and the fed (1.8 h) groups. The oral bioavailability *F*-values were found to be 3.7% (fasted) and 3.8% (fed), indicating no influence of food on the bioavailability of IQG-607 (Dadda et al., 2018). The low oral bioavailability F observed in mice could be related to a high first pass effect following oral dosing, among other factors such as subtract to efflux transporters or low gastrointestinal permeability (Kwan, 1997; Martinez and Amidon, 2002) which should be investigated. The oral bioavailability determined for IQG-607 cannot be used to rule out this anti-TB candidate because there are various drug candidates and also commercially available drugs that exhibit low oral bioavailability, such as SQ109 (*F* = 4%) (Jia et al., 2005) and buspirone (F = 3.9%) (Brown and Tomlin, 2010). Several studies show that other determinants for the clinical outcome of a chemical compound have to be taken into consideration such as the drug candidate levels at the target site (Ryan, 1993; Presant et al., 1994; Joukhadar et al., 2001; Jia et al., 2005). Preclinical studies of INH pharmacokinetics in mice (both i.v. and oral) are lacking to allow comparisons to be made with the results here presented. Reported data show that INH is rapidly absorbed in mice (Grosset and Ji, 1998), with a *t*_max_ value similar to IQG-607 (0.25 h) (Dadda et al., 2018). Interestingly, the *t*_1/2_ value of IQG-607 after oral administration (1.6 and 1.3 h for fasted and fed mice, respectively) (Dadda et al., 2018) was also similar to INH (1.7 h) (Grosset and Ji, 1998). In addition, it is known that some types of foods, especially carbohydrates, can affect the oral bioavailability of INH (Männistö et al., 1982; Peloquin et al., 1999; Self et al., 1999), which has not been observed for IQG-607 (Dadda et al., 2018). In addition; a value of 88.9 (± 0.9%) for IQG-607 protein binding in mice plasma, which was independent of compound concentration, was determined by ultrafiltration (Dadda et al., 2018).

The developed HPLC method was adapted to quantify IQG-607 in minipigs plasma to determine the pre-clinical pharmacokinetic parameters (non-compartmental analysis). After oral administration of IQG-607 (220 mg/kg), the levels of the compound in minipigs plasma were quantified at time points for up to 7 h (Rodrigues-Junior et al., 2017a). The *C*_max_ (0.8 μg/mL) was achieved after 1 h (*t*_max_). The AUC_0 → *t*_ and AUC_0 → ∞_ were, respectively, 3.0 and 5.9 μg/L h. The Ke and *t*_1/2_ values were, respectively, 0.14 h^−1^ and 5.8 h, while *CL* and MRT values were 39 L/h kg and 33 h, respectively (Rodrigues-Junior et al., 2017a). The values of pharmacokinetic parameters of minipigs were higher than those of mice, except *C*_max_ and Ke (0.8 μg/mL and 0.14 h^−1^, respectively, for minipigs; and 1.23 μg/mL and 0.43 h^−1^, respectively, for mice). As data for i.v. administration of IQG-607 in minipigs are not yet available, it is not possible to calculate the oral bioavailability F because the AUC i.v. needs to be obtained to apply the following equation: [%F = (AUC_0 → ∞(*po*)_/AUC_0 → ∞_(iv)) (Dose_(i.v.)_/Dose_(po)_)100%]. AUC_0 → ∞(*po*)_ represents area under the curve from time zero to infinity after oral administration and AUC_0 → ∞(*i.v*.)_ represents area under the curve from time zero to infinity after i.v. administration, Dose_(po)_ is the dose administered orally, and Dose_(i.v.)_ is the dose administered i.v. (Shargel et al., 2005). Unfortunately, there are no preclinical studies of INH in minipigs available to allow comparisons to be made with the results for IQG-607. The data present here should serve as support for future pre-clinical pharmacokinetic/pharmacodynamic modeling studies, and in the study of different formulations to improve the bioavailability of IQG-607, if proved necessary.

## Elucidation of mechanism of *M. tuberculosis* resistance to IQG-607

As mentioned above, the IQG-607 compound was rationally designed to be active against INH-resistant strains of *M. tuberculosis*. Two alternate mechanisms were put forward to explain the mode of action of IQG-607 and its likely effect on INH-resistant *M. tuberculosis* strains. On one hand, a possible explanation involved a redox-mediated self-activation of the inorganic complex within the mycobacteria by reactive oxygen species (e.g., hydrogen peroxide and superoxide) coordinated by the pentacyanoferrate(II) metal center forming the pentacyanoINH-NAD adduct, which would thereby bypass the need for KatG activation (Sousa et al., 2014; Laborde et al., 2018). On the other hand, enzymatic inhibition assays showed that it was capable of inactivating both wild-type InhA enzyme and proteins harboring *inhA* gene mutations in the absence of NADH and without requiring KatG activation (Oliveira et al., 2004; Vasconcelos et al., 2008). These latter results suggested that the compound was not a pro-drug candidate and that it would probably interact directly with the NADH binding site of the enzyme, without forming an adduct with NAD. Although these two mechanisms would follow different pathways, both were proposed to avoid the requirement for KatG activation and, ultimately, kill strains carrying mutations in its coding sequence.

To try to shed light on the mechanism of resistance of *M. tuberculosis* to IQG-607, its inhibitory activity against MDR-TB clinical isolates harboring the KatG(S315T) mutation was evaluated (the description of *inhA* promoter, *katG* and *rpoB* gene mutations of these isolates are described in Table 1 of Abbadi et al., 2018). Surprisingly, eight of the nine clinical isolates tested were resistant to both IQG-607 and INH, with MIC values larger than 100 μg/mL (Abbadi et al., 2018). These results were unexpected and challenged both mechanisms of action previously proposed for this compound, since it suggested an association between the presence of the S315T mutation found in the eight clinical isolates and increased MIC values (>64-fold) for IQG-607, implying that KatG activation is needed for its inhibitory activity. To further investigate if the mechanism of resistance to IQG-607 would resemble that observed for INH, spontaneous drug-resistant mutants were isolated using concentrations larger than the MIC value for IQG-607, followed by whole-genome sequencing. Similar experimental strategies have been successfully used for target identification of novel anti-TB compounds (Lechartier et al., 2014), such as bedaquiline (Andries et al., 2005), pyridomycin (Hartkoorn et al., 2012), and Q203 (Pethe et al., 2013), and for the identification of non-essential enzymes that act as activators of prodrugs as isoniazid (Bergval et al., 2009), ethionamide (Brossier et al., 2016) and pyrazinamide (Stoffels et al., 2012). Whole-genome and targeted DNA sequencing of six resistant colonies showed that all of them harbored alterations in the *katG* gene, thereby suggesting that IQG-607 needs to be activated by *M. tuberculosis* KatG enzyme. Four colonies had single deletions in the gene-coding sequence that led to frameshift mutations (Abbadi et al., 2018), a genetic profile that is not common in clinical isolates. These findings were in agreement with previous reports using INH to isolate *in vitro* spontaneous mutants (Bergval et al., 2009; Brossier et al., 2016). It is believed that the high proportion of INH-spontaneous mutants having a defective *katG* gene is due to the favorable culture conditions in the laboratory, which barely represent the oxidative stress that mycobacteria face inside the host macrophages. Hence, in the secure environment of the culture medium, the protective role displayed by KatG against oxidative burst would not be required (Manca et al., 1999; Brossier et al., 2016). Two colonies harbored missense mutations in the *katG* gene (W91R and H417D), providing further support for KatG activation of IQG-607 (Abbadi et al., 2018). The W91R KatG mutant was described at low frequency in clinical isolates (Ramaswamy et al., 2003; Vilchèze and Jacobs, 2014), while the H417D KatG mutant had not been previously reported. Both mutations caused co-resistance to INH and IQG-607 according to MIC determination assays (Abbadi et al., 2018). It was proposed that the Trp-91 residue participates in an important H-bonding network into KatG's active site, together with the Trp-107 of the Met-255, Tyr-229 and Trp-107 cross-link (Met-Tyr-Trp), a structural feature in which three non-sequential residues are covalently linked together through their side chains, several water molecules, the heme propionate arm, and the distal His-108 and Arg-104 residues (Ghiladi et al., 2005). Hence, the replacement of the redox active Trp-91 by an arginine residue could disrupt this H-bonding network that is involved in INH oxidation, and which may tentatively be extended to IQG-607 oxidation process. Although the role of the His-417 residue is still not known, a neighboring residue (Arg-418) plays an important role in catalase activity (Mo et al., 2004; Ghiladi et al., 2005; Cade et al., 2010). The Arg-418 residue forms two hydrogen bonds with the Met-Tyr-Trp crosslink of KatG and mutations in this residue are known to cause INH resistance (Ghiladi et al., 2005).

The next step for elucidating the mechanism of resistance to IQG-607 was to establish a causal relationship between the presences of the S315T mutation with the resistant phenotype against this compound. It is known that mutations in this residue do not affect catalase and peroxidases activities, but cause INH resistance by constricting the substrate access channel of the enzyme (Zhao et al., 2006; Cade et al., 2010). The wild-type *katG* gene from *M. tuberculosis* H37Rv strain was disrupted by homologous recombination (Pelicic et al., 1997), and complemented with either wild-type or the mutant allele (S315T). This mutation was capable of increasing in more than 64-fold the MIC value for IQG-607 (Abbadi et al., 2018), suggesting that IQG-607 requires KatG activation for its inhibitory activity. As one of the proposed mechanism for IQG-607 required redox-mediated activation in the oxidizing environment of macrophages, isoniazid-based radical formation without KatG assistance, followed by adduct formation and InhA inhibition (Sousa et al., 2014), murine macrophages were infected with the *katG*(S315T) mutant strain. The results showed that the self-activation of IQG-607 was not triggered by the host intracellular environment, as the mutant strain maintained its resistant phenotype in the presence of the compound (Abbadi et al., 2018). Taken together, these results strongly suggest that IQG-607 acts as a prodrug that requires KatG enzyme activation to exhibit *M. tuberculosis* growth inhibition.

The target of IQG-607 was also genetically validated (Abbadi et al., 2018). Expression of wild-type InhA did not increase the MIC values for INH and ETH, as would be expected, but raised in 2-fold the MIC value for IQG-607 (Abbadi et al., 2018). It is likely that the lack of resistance to INH and ETH is due to low expression level of the wilt-type InhA enzyme (Abbadi et al., 2018). It is known that the S94A mutant of InhA protein confers *in vitro* resistance to INH in *M. tuberculosis* (Vilchèze et al., 2006). Overexpression of S94A InhA mutant enzyme in *M. tuberculosis* cells resulted in resistance to IQG-607 (Abbadi et al., 2018), suggesting that InhA is the main target of this compound. This result is in agreement with abrogation of mycolic acid biosynthesis by IQG-607 as assessed by incorporation of radiolabelled precursor acetate (Rodrigues-Junior et al., 2014). Interestingly, the data for S94A InhA mutant expression in *M. tuberculosis* were not expected as IQG-607 was shown to inhibit the activity of this mutant protein *in vitro* (Vasconcelos et al., 2008). Similar results were observed for INH. The inhibition parameters for the InhA S94A mutant protein were shown to be comparable to those seen for the wild-type enzyme *in vitro* (Rawat et al., 2003). However, it is sufficient to confer resistance to INH in the intracellular context (Vilchèze et al., 2006), in which the enzyme may be assembled into a multienzimatic complex with other proteins of the FAS-II system (Cantaloube et al., 2011). Accordingly, only in the context of intracellular mycolic acid biosynthesis interactome (Cantaloube et al., 2011) would S94A mutant protein cause an INH-resistant phenotype as *in vitro* assays are carried out with recombinant homogeneous isolated InhA. Future experiments will be necessary to unveil the metabolites formed by IQG-607 activation to define the chemical compound that inhibits InhA enzyme activity in *M. tuberculosis* cells. These results also highlighted the challenges involved in translating conclusions based on *in vitro* experiments to the intracellular environment of *M. tuberculosis*, let alone its interactions with the human host.

## Perspectives (nanodelivery systems or nano-formulations)

The favorable safety profile for IQG-607 in cells, mice, rats, and minipigs (Basso et al., 2010; Amorim et al., 2017; Rodrigues-Junior et al., 2017a,b) suggest that further efforts may be worth pursuing. Moreover, the reduced CFU counts in the lungs and spleens of *M. tuberculosis*-infected mice treated with IQG-607 suggest that this metal-based compound is absorbed after oral administration, can reach the lungs, and kill the bacilli in the phagosome of macrophages. Drug associations should be investigated for HIV co-infected patients under high potency antiretroviral therapy that present severe drug interactions. Efforts should also be pursued to determine the metabolites formed by IQG-607 activation to define the chemical compound that inhibits InhA enzyme activity in *M. tuberculosis* cells. Comparative toxicological studies are currently underway to investigate if IQG-607 may represent an alternative to INH causing less drug-induced liver injuries.

Although a number of parameters should be taken into account to move a compound from the hit list to approved drug, the short half-live and low oral bioavailability F observed in mice for IQG-607 (section Methods for Quantification and Pharmacokinetic Studies) may deserve more attention before moving forward. A number of factors could be invoked to account for the low oral bioavailability F of IQG-607, for instance: low gastrointestinal permeability, first pass metabolism, distribution and biotransformation that limit the dose at the target site, and efflux pumps. Moreover, the relationship between concentrations of drugs in the systemic circulation, in the epithelial lining fluid, and tissue mass in different lobes of the lung has to be taken into account as the drug must penetrate from the systemic circulation to the granulomatous lesions where the mycobacteria exist (Gaohua et al., 2015). Accordingly, several physiological barriers need to be overcome when drugs are orally administered, including first-pass metabolism, adequate permeability in lung and uptake into *M. tuberculosis* to reach the intracellular target(s) (Dartois, 2014). Furthermore, chemical stability under different physiological conditions of the multicellular structures that are characteristics of the TB pathology, such as necrotizing or caseum granulomas must be considered (Dartois and Barry, 2013; Sarathy et al., 2018).

The development of technological formulations may be a way forward to ensure that therapeutic concentrations of IQG-607 are achieved in multiple types of injuries and to increase the ability of this compound to penetrate and kill the different populations of *M. tuberculosis* in infectious injuries (intra- and extracellular, replicating or dormant TB). Currently, one promising approach to the development of innovative and effective formulations for the delivery of anti-TB drugs is nanotechnology (Costa et al., 2016; Costa-Gouveia et al., 2017). Colloidal nanocarriers are nanoparticle systems of submicron particle size (≤1,000 nm) composed by several types and morphologies, such as nanocapsules, polymer nanoparticles, liposomes, polymerized micelles and others (Griffiths et al., 2010; Kaur and Singh, 2014). Different types of nanocarriers are designed to coat ultra-dispersed drugs and to take them to specific intracellular targets which are surrounded by complex physiological barriers, providing a protective effect (Desjardins and Griffiths, 2003). Nanocarriers are thus an attractive tool for the internalization of the drug in the infected macrophages because they can internalize particles up to 10 μm and with a wide range of receptors present on its surface by phagocytosis (Areschoug and Gordon, 2009). Other potential advantages of the nanosystem drugs include the possibility of alternative routes of administration for the treatment of TB: pulmonary (Maretti et al., 2014; Costa-Gouveia et al., 2017; Salzano et al., 2017), subcutaneous (Pandey and Khuller, 2004), intravenous (Saraogi et al., 2011), and oral (Pandey et al., 2003; Sharma et al., 2004). Pre-clinical studies have shown that nanodelivery systems, when controlling the delivery of the first-line as well as second-line anti-TB drugs, offer potential advantages over free drugs (Gangadharam et al., 1991; Costa-Gouveia et al., 2017). The advantages of nanodelivery systems include targeting of specific tissues and cells infected with *M. tuberculosis*, thus increasing their therapeutic efficacy, decreasing systemic toxicity and prolonging drug release, allowing the use of less frequent doses (Anisimova et al., 2000; Pandey et al., 2003; Sharma et al., 2004). Others sophisticated nanocarriers have been developed to optimize loading and release of high concentrations of intracellular antituberculosis drugs. For example, mesoporous silica nanoparticles (MSNP) loaded with rifampicin or isoniazid and equipped with pH-operated nanovalves showed an ability to kill the *M. tuberculosis* into macrophages more efficiently than an equivalent amount of free drugs (Clemens et al., 2012). Multifunctional nano-structured carriers using graphene oxide with iron oxide nanoparticles magnetite have successfully been designed for controlled delivery of ethambutol and potent antituberculosis activity, remaining non-toxic to the eukaryotic cells tested (Saifullah et al., 2017).

IQG-607 has relatively fast oral absorption and elimination, and high plasma protein binding (Dadda et al., 2018). However, its low toxicity opens new possibilities for the treatment of TB. Therefore, the short half-life and the low oral bioavailability highlight an opportunity for innovative research in nanodelivery systems as a strategy to enhance *in vivo* efficacy and to improve its bioavailability. On the other hand, as IQG-607 is a therapeutic structural analog of INH some important disadvantageous aspects of the INH therapy should be considered: prolonged treatment duration and higher rates of treatment failure in noncompliance patients; negative pharmaceutical interactions; toxic side effects; and the emergence of INH resistance (Preziosi, 2007). Furthermore, INH is metabolized by arylamine *N*-acetyltransferase 2 (NAT2) lightly contributing to the resistance of the TB treatment in isolated clinical cases and INH-induced hepatotoxicity (Evans et al., 1960; Pasipanodya et al., 2012). Considering these clinically relevant aspects, nanodelivery systems could also be employed to controlled-release of IQG-607 to the target site and could potentially prevent the hepatotoxic effects associated with the genetic polymorphism of NAT2 in humans. Moreover, IQG-607-loaded nanosystems can ensure resistance to possible acetylation of the pyridine-4-carbohydrazide moieties, increasing the residence time of IQG-607 in the blood circulation as well as protecting it from both possible enzymatic and the physico-chemical degradations, probably resulting in reduction of therapeutic concentration and dosing frequency.

It should be noted that recently reported data suggest that IQG-607 requires “bioactivation” by mycobacterial KatG. However, from the route of administration to infection site many redox reactions may cause a change in the oxidation state of the central metal ion before reaching its target site. Therefore, a better understanding of IQG-607 mechanism of action and identification of its chemical species formed by chemical redox reactions and enzyme metabolism are crucial for the design of nano-formulations that would impart long half-life in the physiological environment thereby improving its bioavailability.

**Figure 6:**
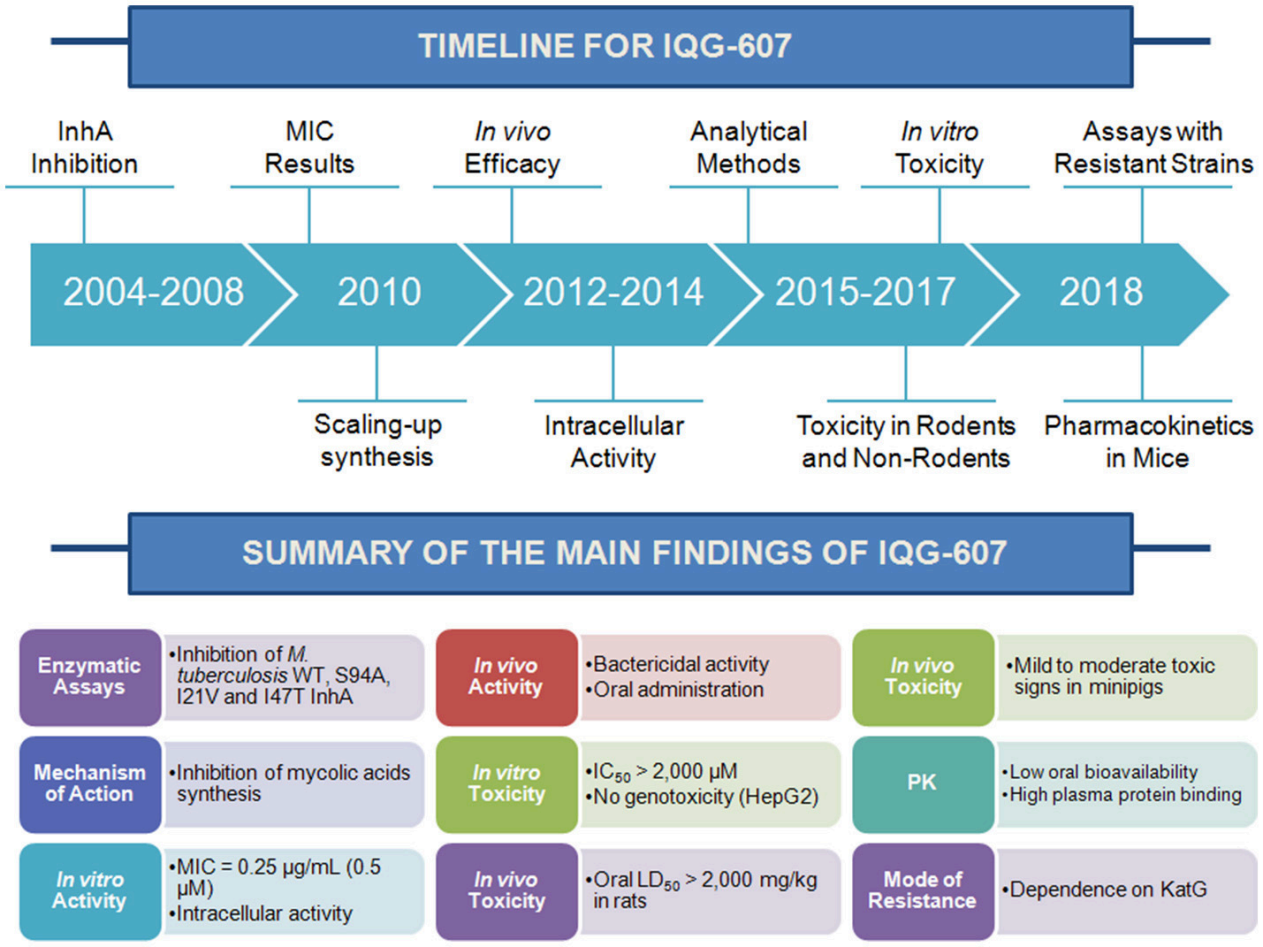
Timeline for IQG-607.

The genetic results described in the section Elucidation of Mechanism of *M. tuberculosis* Resistance to IQG-607 suggest that IQG-607 acts as a metallopro-drug that requires KatG enzyme activation to form a yet unknown inhibitor of InhA activity, which leads to abrogation of mycolic acid biosynthesis and *M. tuberculosis* growth inhibition. These results appear to answer, at least partially, the question posed in the title of our contribution. For last, a timeline for IQG-607 giving a summary of the main experimental findings is given in Figure 6.

## Author contributions

All authors contributed conception and design of the studies described. All authors contributed to writing separate sections of the first draft and manuscript revision. All authors read and approved the submitted version.

### Conflict of interest statement

The authors declare that the research was conducted in the absence of any commercial or financial relationships that could be construed as a potential conflict of interest. The reviewer CFZ and handling Editor declared their shared affiliation.

## Abbreviations

AUC_0 → *t*_: area under the concentration-time curve from 0 to time *t*
AUC_0 → ∞_: area under the plasma concentration–time curve from time zero to infinity
CFU: colony forming units
*CL*: clearance
*C*_max_: maximum (peak) concentration in plasma
DEPMPO: 5-(diethoxyphosphoryl)-5-methyl-1-pyrroline-N-oxide
DMSO: dimethylsulfoxide
ETH: ethionamide
F: oral bioavailability
FDMPO, 4-hydroxy-5: 5-dimethyl-2-trifluoromethylpyrroline-1-oxide
INH: isoniazid
InhA: 2-*trans*-enoyl-ACP(CoA) reductase from *Mycobacterium tuberculosis*
IQG-607: Na_3_[Fe^II^(CN)_5_(INH)]·4H_2_O, pentacyano(isoniazid)ferrate(II) complex
KatG: catalase peroxydase from *Mycobacterium tuberculosis*
Ke: elimination rate constant
LD_50_: median lethal dose
MABA: colorimetric microplate resazurin-based Alamar Blue assay
MDR-TB: multidrug-resistant TB
MIC: minimum inhibitory concentration
MRT: mean residence time
PBN: N-tert-butyl-α-phenylnitrone
POBN: α-(4-pyridyl-N-oxide)-N-tert-butylnitrone
PZN: pyrazinamide
ROS: reactive oxygen species
RR-TB: rifampicin-resistant TB
SI: selective index (SI = IC_50_/MIC)
*t*_1/2_: half-life
*t*_max_: time to reach *C*_max;_ TB,tuberculosis
Vdss: volume of distribution at steady state.
